# The reduction of race and gender bias in clinical treatment recommendations using clinician peer networks in an experimental setting

**DOI:** 10.1038/s41467-021-26905-5

**Published:** 2021-11-15

**Authors:** Damon Centola, Douglas Guilbeault, Urmimala Sarkar, Elaine Khoong, Jingwen Zhang

**Affiliations:** 1 Annenberg School for Communication, University of Pennsylvania, Philadelphia, PA 19106 USA; 2 School of Engineering, University of Pennsylvania, Philadelphia, PA 19106 USA; 3 Department of Sociology, University of Pennsylvania, Philadelphia, PA 19106 USA; 4 Network Dynamics Group, University of Pennsylvania, Philadelphia, PA 19106 USA; 5 Hass School of Management, University of California, Berkeley, Berkeley, CA 94720 USA; 6 Division of General Internal Medicine, University of California, San Francisco, San Francisco, CA 94110 USA; 7 Department of Communication, University of California, Davis, Davis, CA 95616 USA

**Keywords:** Diagnosis, Sociology

## Abstract

Bias in clinical practice, in particular in relation to race and gender, is a persistent cause of healthcare disparities. We investigated the potential of a peer-network approach to reduce bias in medical treatment decisions within an experimental setting. We created “egalitarian” information exchange networks among practicing clinicians who provided recommendations for the clinical management of patient scenarios, presented via standardized patient videos of actors portraying patients with cardiac chest pain. The videos, which were standardized for relevant clinical factors, presented either a white male actor or Black female actor of similar age, wearing the same attire and in the same clinical setting, portraying a patient with clinically significant chest pain symptoms. We found significant disparities in the treatment recommendations given to the white male patient-actor and Black female patient-actor, which when translated into real clinical scenarios would result in the Black female patient being significantly more likely to receive unsafe undertreatment, rather than the guideline-recommended treatment. In the experimental control group, clinicians who were asked to independently reflect on the standardized patient videos did not show any significant reduction in bias. However, clinicians who exchanged real-time information in structured peer networks significantly improved their clinical accuracy and showed no bias in their final recommendations. The findings indicate that clinician network interventions might be used in healthcare settings to reduce significant disparities in patient treatment.

## Abstract (web summary)

Race and gender bias in healthcare contribute to health disparities. Here the authors show in an experimental setting that structured information sharing networks among clinicians can reduce race and gender bias in medical decisions.

## Introduction

Bias is an enduring cause of healthcare disparities by race and gender^1–7^. Previous experimental work demonstrated that clinicians reviewing video-based vignettes of high-risk patients with chest pain disproportionately referred men compared to women, and white patients compared to Black patients for the guideline-recommended treatment, cardiac catheterization^1,7^. Proposed solutions for addressing bias have focused on cognitive strategies that increase clinicians’ awareness of their own biases^6,8–11^. However, no approaches have yet been found that successfully reduce race and gender bias in clinical treatment recommendations^6,10^.

Recent research in non-clinical settings has shown that information exchange in large social networks with uniform—i.e. egalitarian^12^—connectivity can be effective for improving collective intelligence in both health-related and non-health-related risk assessments^13–16^. Studies of bias reduction in partisan networks^15,16^ have found that this process of collective learning in egalitarian networks can effectively reduce, and even eliminate longstanding political biases in the evaluation of novel information^16^. Here, we integrate this recent work on bias reduction in egalitarian information-exchange networks with theoretical research on medical reasoning^17–19^, which has argued that improving the accuracy of clinicians’ diagnostic assessments should improve the quality of their treatment recommendations^19–21^. We hypothesize that creating structured information-exchange networks among clinicians will lead to improved clinical assessments that may be effective for reducing observed patterns of race and gender bias in clinical treatment recommendations^13,14^. Despite the broad practical^2–4^ and scientific^1,5–7^ importance of understanding and addressing bias in medical settings, it has not been possible to evaluate this hypothesis because such a test requires the ability to experimentally isolate and measure the direct effects of clinical networks on reducing medical bias and treatment disparities.

We adopted an experimental approach to evaluate whether large, uniform information-exchange networks among clinicians^13–16^ might significantly reduce observed race and gender bias in clinicians’ treatment recommendations, relative to a control group of independent clinicians who did not participate in information-exchange networks. We recruited 840 practicing clinicians (see Supplementary Information for details) to participate in the online video-based study, which was administered through a proprietary mobile app for clinicians. Each clinician viewed a standardized patient video of either a white male patient-actor or Black female patient-actor, and provided clinical assessments and treatment recommendations for the depicted clinical case (see SI, Supplementary Fig. 5 and Supplementary Fig. 6). Both the white male and Black female “patients” in the videos were portrayed by professional actors who appeared 65 years old, were dressed in identical attire, and depicted a patient with clinically significant chest pain symptoms. The actors portraying each patient followed a single script, in which they provided an identical clinical history that included several risk factors for coronary artery disease (age, hyperlipidemia, and discomfort with exertion). Both videos were accompanied by an identical electrocardiogram exhibit showing abnormalities. (Hereafter, we refer to the patient-actors in the standardized patient videos as “patients”.)

After viewing the patient video, clinicians were asked to provide their initial clinical assessments and treatment recommendations. Clinical assessments took the form of a probability estimate (from 0 to 100) of the patient’s chance of having a major adverse cardiac event within the next 30 days. The most accurate assessment based on the patient’s HEART score is 16%^22,23^. (Additional analyses show the robustness of our findings across a range of assessment values. See SI “Sensitivity Analyses”, Supplementary Fig. 9 and Supplementary Fig. 10). Clinicians then selected a single treatment recommendation from four multiple choice options: Option A. daily 81 mg aspirin and return to clinic in one week (i.e. unsafe undertreatment); Option B. daily 81 mg aspirin and stress test within two to three days (i.e. undertreatment); Option C. full-dose aspirin and referral to emergency department for evaluation and monitoring (i.e. highest quality, guideline-recommended treatment); or Option D. full-dose aspirin and referral to cardiology for urgent cardiac catheterization (i.e. overtreatment in the context of unconfirmed diagnosis).

Option C is the most appropriate treatment based on currently accepted guidelines from the American College of Cardiology^23,24^ and represents the highest standard of care^22,23^. (In consideration of the fact that some clinicians may choose a less aggressive initial strategy in a patient with atypical symptoms, we conducted sensitivity analyses that accepted Option B and Option C as correct. As reported in Supplementary Fig. 11 and Supplementary Fig. 12 in SI “Sensitivity Analyses”, these analyses show the robustness of our findings for both Option B and Option C). Our primary measure of bias is the rate at which the white male patient versus the Black female patient was given the highest quality, guideline-recommended care (Option C). Our secondary measure of bias is inequity in the treatment of the Black female and white male patients, in terms of the relative rates at which the white male patient and the Black female patient were recommended for unsafe undertreatment (option A) rather than the guideline-recommended care (Option C) (see *SI*). We focus on the relative rates of option A and C because this aligns with the observed racial disparities in workup and referral rates for chest pain in clinical care^25^. Option A is unsafe and inappropriately defers workup to one week later putting the patient at risk of significant adverse outcomes. Option B (undertreatment) is not as unsafe as Option A because it shortens the time period of further evaluation from one week to 3 days; however, it is not the guideline-recommended care, which advises immediate evaluation for cardiac tissue damage to appropriately triage the patient. Option D is incorrect because without a troponin measurement (which assesses for acute damage of cardiac tissue), the patient presentation does not warrant an immediate invasive procedure. Option D exposes the patient to the risk of a potentially unnecessary invasive procedure and wasteful healthcare spending.

In each trial, clinicians were randomized to one of four conditions: (i) network condition with the Black female patient; (ii) network condition with the white male patient; (iii) control (independent reflection) condition with the Black female patient or (iv) control condition with the white male patient. In the two network conditions, clinicians were randomly assigned to a single location in a large, anonymous uniform social network (*n* = 40), in which every clinician had an equal number of connections (*z* = 4), which ensured that no single clinician had greater power over the communication dynamics within the network^13^ (see SI, Supplementary Fig. 4 for network details). Clinicians were anonymous and did not have any information about how many peers they were connected to. Clinicians’ contacts in the network remained the same throughout the experiment. In the two control conditions, clinicians provided their responses in isolation. Because clinicians in the control conditions were independent from one another, fewer overall clinicians were required for the control analyses (*n* = 20 in each trial). For proper comparison with the experimental conditions, we randomly assigned clinicians in each control condition into bootstrapped “groups” of *n* = 40, and conducted our analyses at the group level (see *SI*, “Statistical Analyses”).

In all conditions, clinicians were given three rounds to provide their assessments and treatment recommendations for the presented patient. In the initial round, all clinicians independently viewed their respective videos, and were then given two minutes to provide their clinical assessments and treatment recommendations. In the control conditions, clinicians remained isolated for two additional rounds of evaluation. In round two, they viewed the patient video a second time, and were again given two minutes to respond. Clinicians could either provide the same responses or modify their responses. In the final round, clinicians repeated this procedure again, and provided their final responses. In the network conditions, in round two clinicians were again shown the patient video, as well as being shown the average assessment responses (i.e. diagnostic estimates) of their network contacts, and then asked to provide their assessments and recommendations (see SI, Supplementary Fig. 6). Clinicians could either provide the same responses they gave in the initial round or modify their responses. In the final round, this procedure was repeated again showing the average responses from round two, and clinicians were asked to provide their final responses.

Each trial lasted ~8 min. Participants’ compensation was based on their performance in the final round. Only clinicians who provided the guideline-recommended clinical recommendation in their final responses were given a payment of $30. Clinicians who provided other responses were not compensated for their participation. 86% of participating clinicians completed our study. (Analyses provided in the SI show that all of our results are robust to the inclusion or exclusion of attrited participants, see SI “Sensitivity Analyses”).

We conducted seven independent trials of this study from March 1, 2019 to November 29, 2019. Except where explicitly noted, all statistical analyses were conducted at the trial level (*n* = 7 trials × 4 experimental conditions = 28 trial-level observations). The conservative statistical approach we adopt here (reporting trial-level observations) reduces our power to detect effects of the experimental intervention, but it controls for the nonindependence among clinicians in the network conditions, enabling the direct comparison of each trial-level observation across all four experimental conditions. When individual-level analyses are presented using regression techniques, all standard errors are clustered at the trial level to preserve trial-level comparisons. (Additional analyses in the SI show that our findings are confirmed, and significantly strengthened using individual-level regression analyses with clustered standard errors. See SI, Supplementary Tables 6 to 14). Except for the presence of peer information in the network conditions, participant experience was identical across all experimental conditions. Consequently, any significant differences across experimental conditions in the change in clinicians’ treatment recommendations (from initial to final response) can be attributed to the direct effects of peer interaction networks on clinicians’ decision-making.

## Results

We now present the results indicating the effects of social networks on clinicians’ revisions to their diagnostic assessments and their treatment recommendations. In the following analyses, diagnostic accuracy is defined as the absolute number of percentage points between a clinician’s diagnostic assessment and the most accurate diagnostic assessment. For clarity of presentation, we normalize diagnostic accuracy on a 0–1 scale by applying min-max normalization to the absolute error of clinicians’ diagnostic assessments. Under this procedure, the minimum possible accuracy (indicated by 0) corresponds to the diagnostic assessment with the greatest absolute error (i.e. an estimate that is as far as possible from the most accurate answer of 16%, which in this case is 84 percentage points), while the maximum possible accuracy (indicated by 1) corresponds to a diagnostic assessment that is 0 percentage points away from the most accurate answer, such that they are equivalent (SI, “Statistical Analyses”). As above, in the discussion of our results we refer to the patient-actors in the standardized patient videos as “patients”.

### Initial race and gender bias

**Fig. 1:**
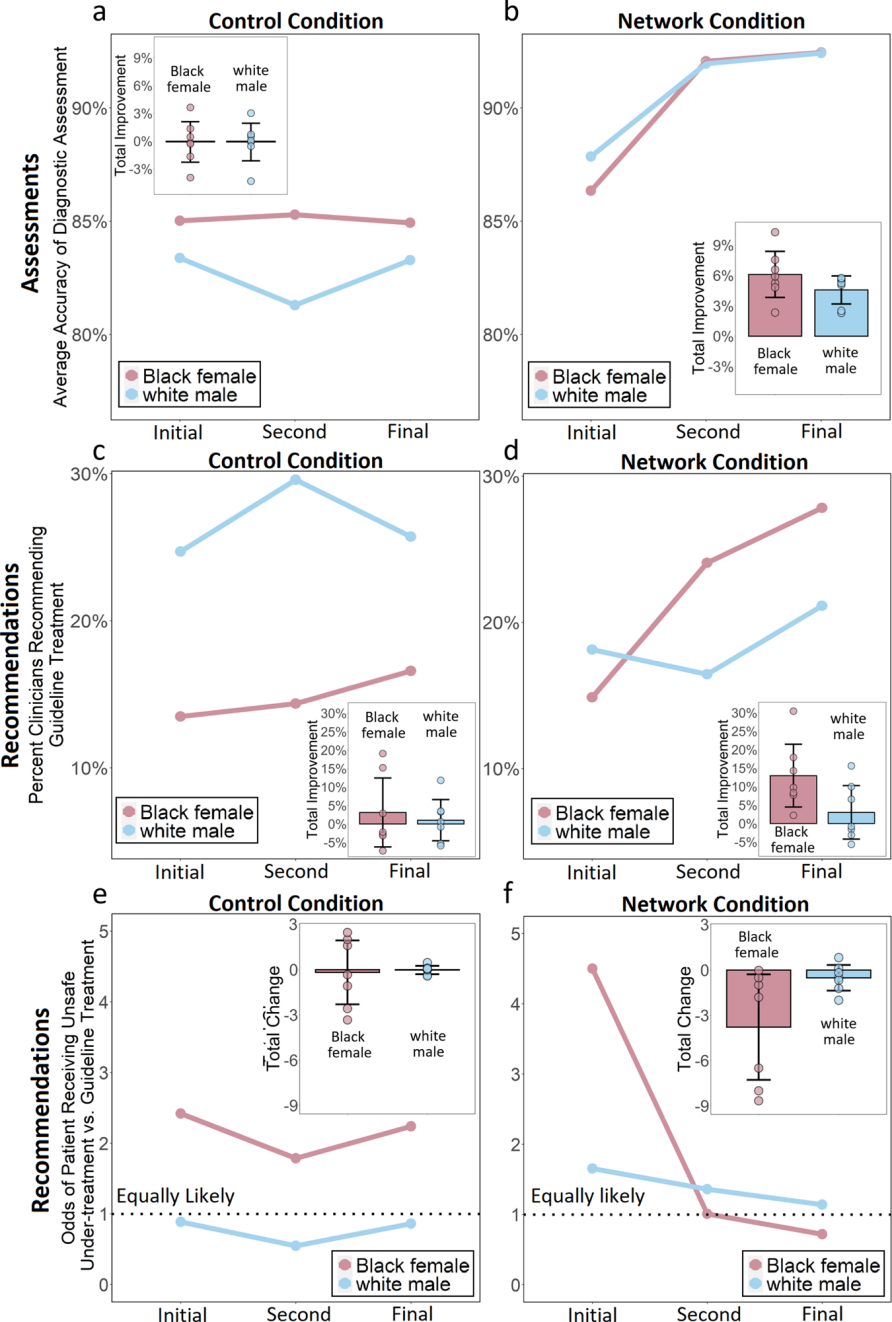
Changes in clinicians’ diagnostic assessments and treatment recommendations in the control and the network condition. Panels **a** and **b** show the change (from the initial assessment to the final assessment) in the average diagnostic accuracy of clinicians. Panel **a** shows the control conditions. Panel **b** shows the network conditions. The insets in both panels show the total improvement (in percentage points) in the accuracy of clinicians’ diagnostic assessments. Error bars display 95% confidence intervals; data points display the mean change for each of the trials (*N* = 7) in each condition. Panels **c** and **d** show the change (from the initial recommendation to the final recommendation) in the proportion of clinicians recommending the guideline-recommended treatment recommendation—referral to the emergency department for immediate cardiac evaluation (Option C)—for the white male patient-actor and Black female patient-actor. Panel **c** shows the control conditions. Panel **d** shows the network conditions. The insets in both panels show the total improvement (in percentage points) in the percent of clinicians recommending the guideline-recommended treatment. Error bars display 95% confidence intervals; data points display the mean change for each of the trials (*N* = 7) in each condition. Panels **e** and **f** show the change (from the initial response to the final response) in the odds of clinicians recommending option A (unsafe undertreatment) rather than option C (highest quality, guideline-recommended treatment) for each patient-actor. Panel **e** shows the control conditions. Panel **f** shows the network conditions. The insets in both panels show the total reduction in the likelihood that clinicians would recommend unsafe undertreatment rather than the guideline-recommended treatment for each patient-actor. Error bars display 95% confidence intervals; data points display the mean change for each of the trials (*N* = 7) in each condition.

Clinicians’ initial assessments and treatment recommendations were made independently. Figure 1 shows that for the initial responses of all clinicians in the study, there were no significant differences in the accuracy of the diagnostic assessments (Fig. 1a, b) given to the Black female patient and the white male patient (*p* > 0.5, *n* = 28, Wilcoxon Rank Sum Test, Two-sided); nor were there any significant differences in the accuracy of initial diagnostic assessments when controlling for experimental condition using a regression approach (β = 1.06, CI = [−3.79 to 5.92], *p* = 0.67, Supplementary Table 6). However, consistent with previous studies of bias in medical care^2–6^, despite clinicians providing both patients with similar diagnostic assessments, clinicians’ treatment recommendations varied significantly between patients. Across all clinicians, their initial treatment recommendations (Fig. 1c, d) show a significant disparity in the rate at which the guideline-recommended treatment was recommended for the white male patient versus the Black female patient. Overall, clinicians recommended Option C, referral to the emergency department for immediate evaluation, for the white male patient in 22% of responses, while only making this recommendation for the Black female patient in 14% of responses (*p* = 0.02, *n* = 28 observations, Wilcoxon Rank Sum Test, Two-sided).

In the control conditions (Fig. 1a), after two rounds of revision there was no significant change in the accuracy of clinicians’ assessments (i.e. diagnostic estimates) for either the white male patient (*p* > 0.9, *n* = 7, Fig. 1a inset, Wilcoxon Signed Rank Test, Two-sided) or the Black female patient (*p* > 0.9, *n* = 7, Fig. 1a inset, Wilcoxon Signed Rank Test, Two-sided). Correspondingly, Fig. 1c shows that in the control conditions there was no significant change in the rate at which clinicians recommend the guideline-recommend treatment for either the Black female patient or the white male patient (Black female patient showed a 3 percentage point increase, *p* = 0.81, *n* = 7 observations, Wilcoxon Signed Rank Test, Two-sided; white male patient showed a 1 percentage point increase, *p* = 0.93, *n* = 7 observations, Wilcoxon Signed Rank Test, Two-sided; Fig. 1c). Clinicians’ final treatment recommendations in the control conditions still showed a significant disparity between the white male patient and the Black female patient in their rates of referral to the emergency department (*p* = 0.04, *n* = 14 observations, Wilcoxon Signed Rank Test, Two-sided; Fig. 1c).

### Networks reduce race and gender bias

Figure 1b shows that in the network conditions there were significant improvements (from the initial response to the final response) in the accuracy of the assessments given to both the white male patient (*p* = 0.04, *n* = 7, Wilcoxon Signed Rank Test, Two-sided; Fig. 1b inset) and the Black female patient (*p* = 0.01, *n* = 7 observations, Wilcoxon Signed Rank Test, Two-sided; Fig. 1b inset). Figure 1d shows that in the network conditions, after two rounds of revision there was no significant change in the rate at which clinicians recommended the guideline-recommended treatment for the white male patient (*p* = 0.57, *n* = 7 observations, Wilcoxon Signed Rank Test, Two-sided; Fig. 1d inset). This lack of change is due to the fact that, regardless of the accuracy of their initial assessments for the white male patient, clinicians were initially significantly more likely to recommend the guideline-recommended treatment for white male patient (*p* < 0.01, OR = 1.78, CI = [1.2–2.6], Supplementary Table 7). Consequently, improvements in assessment accuracy for the white male patient had a smaller positive impact on increasing clinicians’ likelihood of recommending the guideline-recommended treatment. By contrast, clinicians initially were significantly less likely to recommend the guideline-recommended treatment for the Black female patient (*p* < 0.01, OR = 0.56, CI = [0.38–0.83], Supplementary Table 7), while they were significantly more likely to recommend unsafe undertreatment for this patient (*p* < 0.05, OR = 1.5, CI = [1.08–2.04], Supplementary Table 8). Consequently, improvements in assessment accuracy had a substantially greater effect on the final treatment recommendations for the Black female patient (Fig. 1d). In the network condition, the rate at which clinicians recommended guideline-recommended treatment for the Black female patient increased significantly, from 14% in initial response to 27% in final response (*p* < 0.01, *n* = 7 observations, Wilcoxon Signed Rank Test, Two-sided; Fig. 1d). As a result, clinicians’ final treatment recommendations in the network conditions exhibited no significant disparity between the Black female patient and the white male patient in terms of referral rates to the emergency department (*p* = 0.22, *n* = 14 observations, Wilcoxon Rank Sum Test, Two-sided*;* See Supplementary Table 11).

The primary pathway for bias reduction in the network condition was the effect of improvements in clinicians’ assessment accuracy on reducing the initially high rates at which unsafe undertreatment was recommended for the Black female patient. Figure 1e, f shows the odds of clinicians recommending unsafe undertreatment rather than the guideline-recommended treatment for both patients in both conditions. Consistent with the above discussion, treatment recommendations for the white male patient did not exhibit any bias toward unsafe undertreatment (*p* = 0.19, *n* = 14, Wilcoxon Signed Rank Test, Two-sided). As expected, improvements in assessment accuracy in the network condition did not significantly impact clinicians’ odds of recommending the guideline-recommended treatment rather than unsafe undertreatment for the white male patient (*p* = 0.21, *n* = 7, Wilcoxon Signed Rank Test, Two-sided). By contrast, clinicians initially had significantly greater odds of recommending unsafe undertreatment rather than the guideline-recommended treatment for the Black female patient (Fig. 1e, f; *p* < 0.01, *n* = 28 observations, Wilcoxon Signed Rank Test, Two-sided). Independent revision in the control conditions did not have any impact on the treatment recommendations for either the white male (*p* = 1.0, *n* = 7, Wilcoxon Signed Rank Test, Two-sided) or the Black female patient (*p* = 0.81, *n* = 7, Wilcoxon Signed Rank Test, Two-sided). However, assessment revisions in the network condition led to a significant change in the odds of clinicians recommending the guideline-recommended treatment rather than unsafe undertreatment for the Black female patient (Fig. 1f*p* = 0.01, *n* = 7, Wilcoxon Signed Rank Test, Two-sided). By the final round in the network conditions, there was no significant difference between patients in their odds of having clinicians recommend the guideline-recommended treatment rather than unsafe undertreatment (Fig. 1f, *p* = 0.19, *n* = 14, Wilcoxon Rank Sum Test, Two-sided).

### Network mechanism for bias reduction

**Fig. 2:**
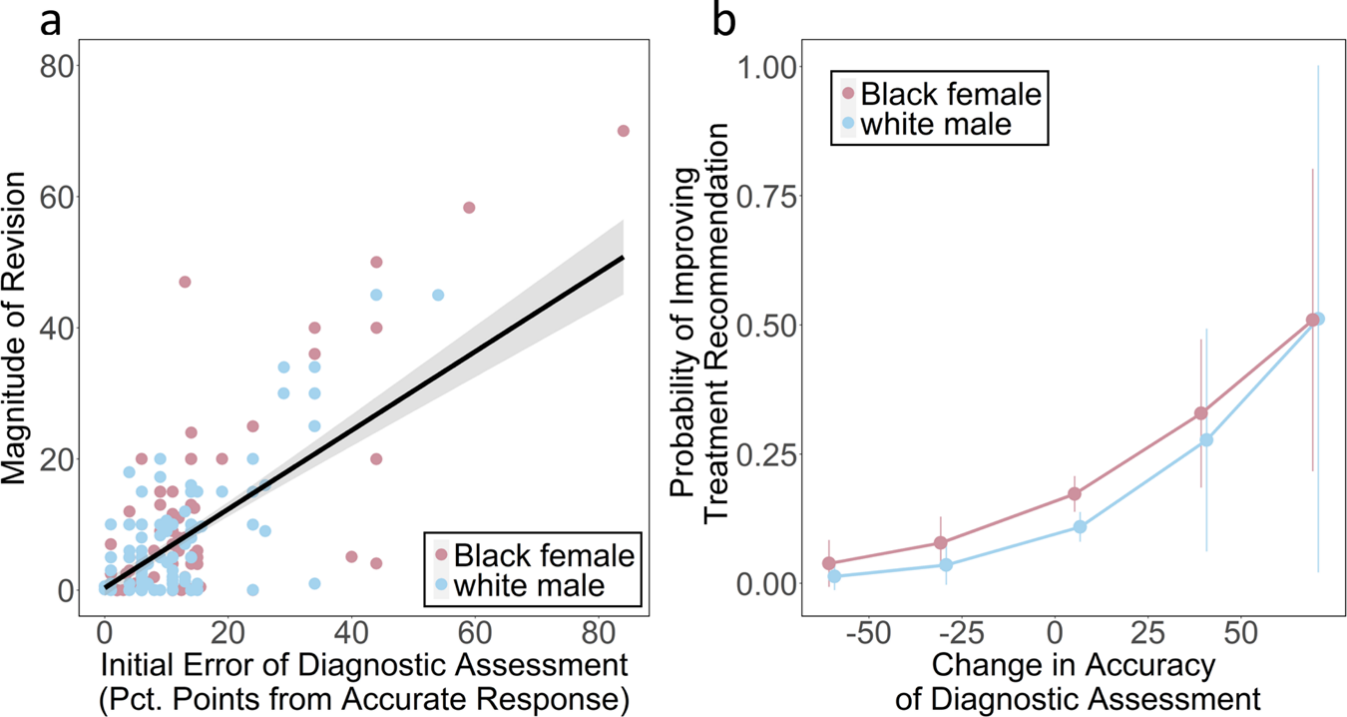
Revision dynamics drive improvements to diagnostic assessments and treatment recommendations. Panel **a** shows clinicians’ propensity to revise their diagnostic assessments in the network conditions according to the initial error in their diagnostic assessments. Clinicians’ accuracy is represented as the absolute number of percentage points of a given assessment from the most accurate assessment of 16% (represented by 0 along the *x*-axis, indicating a distance of 0 percentage points from the most accurate response). Magnitude of revision is measured as the absolute difference (percentage points) between a clinician’s initial diagnostic assessment and their final diagnostic assessment. Clinicians’ accuracy in their initial assessment significantly predicts the magnitude of their revisions between the initial to final response. Grey error band displays 95% confidence intervals for the fit of an OLS model regressing initial error of diagnostic assessment on magnitude of revision. Panel **b** shows the significant positive relationship between the improvement in clinicians’ diagnostic accuracy (from the initial to final assessment), and their likelihood of improving in their treatment recommendation (i.e. the probability of switching from recommending Option A, B, or D to Option C) for clinicians in the network conditions. The trend line shows the estimated probability of clinicians improving their treatment recommendations according to a logistic regression, controlling for an interaction between experimental condition (control or network) and patient-actor demographic (Black female or white male) (Supplementary Table 9). Error bars show standard errors clustered at the trial level.

**Fig. 3:**
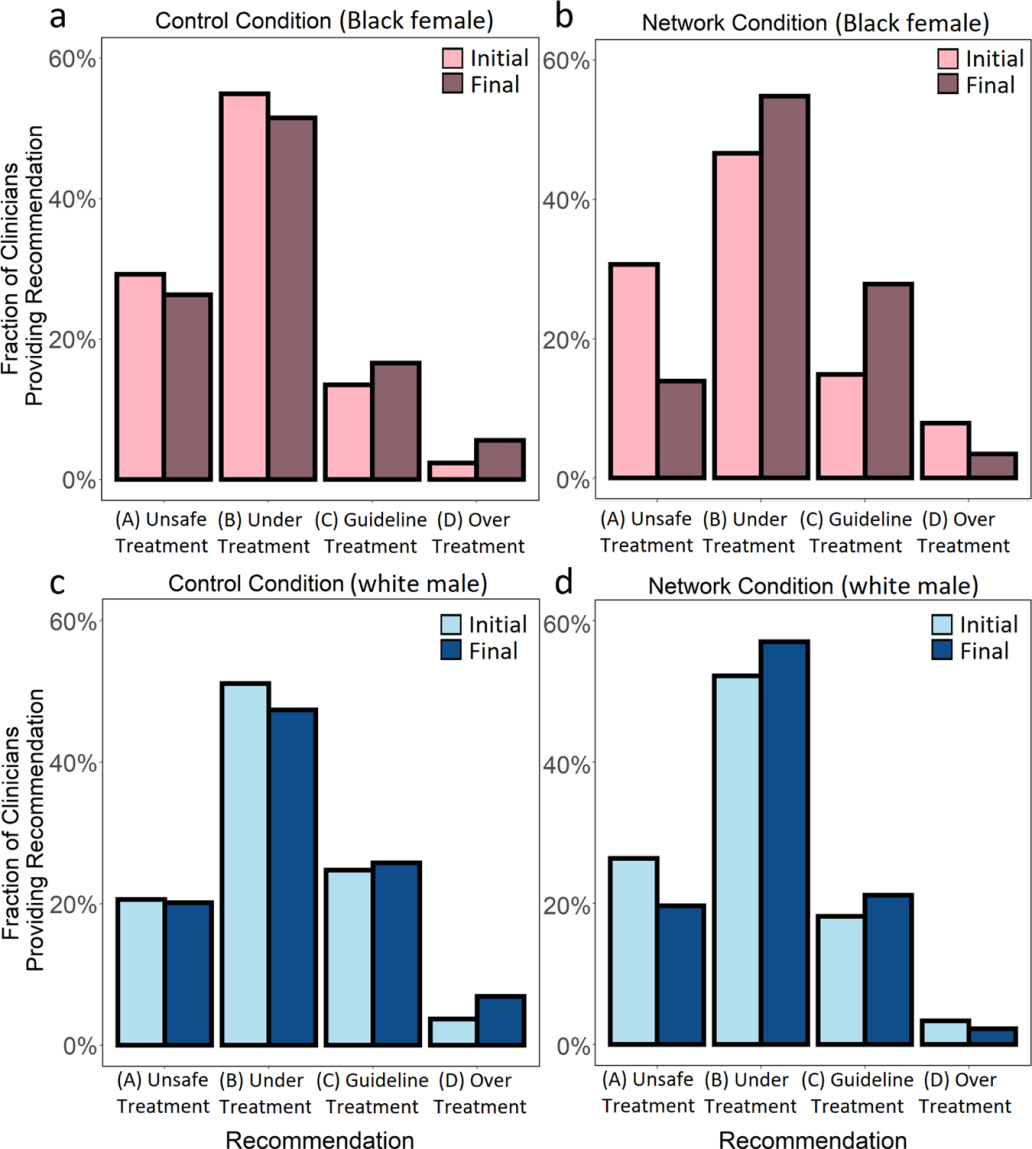
Change (from initial response to final response) in the urgency of clinical recommendations in both the control and network condition. Each panel shows the fraction of clinicians providing each treatment recommendation at the initial and final response, averaged first within each of the trials in each condition (*N* = 7), and then averaged across trials. Option A. 1 week follow-up (unsafe undertreatment). Option B. Stress test in 2–3 days (undertreatment). Option C. Immediate emergency department evaluation (guideline-recommended treatment). Option D. Immediate cardiac catheterization (overtreatment Panel **a** shows the change in control condition recommendations for the Black female patient-actor (initial recommendations light pink, final recommendations dark pink). Panel **b** shows the change in network condition recommendations for the Black female patient-actor (initial recommendations light pink, final recommendations dark pink). Panel **c** shows the change in control condition recommendations for the white male patient-actor (initial recommendations light blue, final recommendations dark blue). Panel **d** shows the change in network condition recommendations for the white male patient-actor (initial recommendations light blue, final recommendations dark blue).

The network mechanism responsible for improvements in the accuracy of clinicians’ assessments, and the corresponding reduction of race and gender disparity in their treatment recommendations, is the disproportionate impact of accurate individuals in the process of belief revision within egalitarian social networks^13,15,16^. As demonstrated in earlier studies of networked collective intelligence^13,15,16^, during the process of belief revision in peer networks there is an expected correlation between the accuracy of an individual’s beliefs and the magnitude of their belief revisions, such that accurate individuals revise their responses less; this correlation between accuracy and revision magnitude is referred to as the “revision coefficient”^13^. Within egalitarian social networks, a positive revision coefficient has been found to give greater de facto social influence to more accurate individuals, which is predicted to produce network-wide improvements in the accuracy of individual beliefs within the social network. These improvements in collective accuracy have been found to result in a corresponding reduction in biased responses among initially biased participants^12,13,15,16^. Figure 2a tests this prediction for clinicians in our study. The results show, as expected, that there is a significant positive revision coefficient among clinicians in the network conditions (*p* < 0.001, *r* = 0.66, SE = 0.1, clustered by trial, Supplementary Table 14), indicating that less accurate clinicians made greater revisions to their responses while more accurate clinicians made smaller revisions, giving greater de facto influence in the social network to more accurate clinicians. This correlation holds equally for clinicians’ assessments for both the white male and Black female patients (Supplementary Table 14). Figure 2b shows that for both patients, improvements in assessment accuracy led to significant improvements in the quality of their treatment recommendations (*p* < 0.05, OR = 1.04, CI = [1.00, 1.09], Supplementary Table 9). Importantly, for clinicians who initially recommended unsafe undertreatment (Option A), we find that improvements in assessment accuracy significantly predict an increased likelihood of recommending the guideline-recommended treatment (Option C) by the final round (*p* < 0.01, OR = 1.17, CI = [1.03, 1.33], Supplementary Table 10). These improvements translated into a significant reduction in the inequity of recommended care for the Black female patient, for whom clinicians were initially significantly more likely to recommend unsafe undertreatment (see Fig. 3, below).

Figure 3 shows the changing rates at which clinicians recommended each option (Option A. unsafe undertreatment, Option B. undertreatment, Option C. guideline-recommended treatment, and Option D. overtreatment) for each patient, from the initial response to the final response, for all conditions. As discussed above, we are particularly interested in the inequity of patient care, defined as the rate at which clinicians made a clearly unsafe recommendation (Option A) versus recommending the guideline-recommended treatment (Option C)^23,24^. Initial responses exhibited significant inequity between patients. Initially, across both conditions, 29.9% of clinicians recommended the unsafe undertreatment for the Black female patient, while only 14.1% recommended the guideline-recommended treatment, resulting in a 15.7 percentage point difference in the rate at which clinicians recommended unsafe undertreatment rather than the guideline-recommended treatment for the Black female patient. By contrast, for the white male patient, 23.4% of clinicians recommended the unsafe undertreatment, while 21.4% of clinicians recommended the guideline-recommended treatment, resulting in a 2 percentage point difference in the likelihood of clinicians recommending unsafe undertreatment rather than the guideline-recommended treatment for the white male patient. This resulted in a 13.7 percentage point difference between the Black female patient and the white male patient in their likelihood of having clinicians recommend unsafe undertreatment rather than the guideline-recommended treatment (*p* = 0.02, *n* = 28 observations, Wilcoxon Rank Sum Test, Two-sided). Individual reflection did not reduce this inequity. The control conditions produced no significant change in the inequity between patients from the initial response to the final response (*p* = 0.57, *n* = 14 observations, Wilcoxon Signed Rank Test, Two-sided). Accordingly, in the final response in the control conditions, there was a 15.3 percentage point difference between the Black female patient and the white male patient in their likelihood of having the clinician recommend unsafe undertreatment rather than the guideline-recommended treatment (*p* = 0.04, *n* = 14 observations, Wilcoxon Rank Sum Test, Two-sided; see SI Eq. 2). Strikingly, however, improvements in diagnostic accuracy in the network condition produced a 20 percentage point reduction in the rate at which clinicians recommended unsafe undertreatment rather than the guideline-recommended treatment the Black female patient (*p* = 0.04, *n* = 14 observations, Wilcoxon Rank Sum Test, Two-sided). By the final response in the network conditions, inequity was eliminated—the Black female patient was no longer more likely than the white male patient to have clinicians recommend unsafe undertreatment rather than the guideline-recommended treatment (*p* = 0.16, *n* = 14 observations, Wilcoxon Rank Sum Test, Two-sided).

### Networks īncrease quality of care for all

Figure 3 (panels a–d) also shows that the network conditions improved the quality of clinical care recommended for both patients (white male and Black female). In particular, for both the Black female and white male patient, the network conditions produced significantly greater reductions in the proportion of clinicians recommending unsafe undertreatment (Option A) than the control conditions (−1.6 percentage point reduction in the control conditions, −11.8 percentage point reduction in the network conditions; *p* < 0.01*, n* = 28 observations, Wilcoxon Signed Rank Test, Two-sided). This reduction in the recommendation of unsafe undertreatment (Option A) was associated with significant increases in recommendations for safer care for both patients. While Option B was not the guideline-recommended treatment, it represents a safer treatment than Option A. Correspondingly, the network conditions significantly increased the proportion of clinicians recommending safer undertreatment (Option B) than the control conditions (−3.5 percentage point reduction in control conditions, +6.5 percentage point increase in the network conditions; *p* = 0.03, *n* = 28 observations, Wilcoxon Signed Rank Test, Two-sided). Strikingly, the rate of overtreatment (i.e. Option D, unnecessary invasive procedure) for both patients was significantly decreased in the network conditions, while it increased in the control conditions (−2.8 percentage point reduction in the network conditions, +3.1 percentage point increase in the control conditions; *p* < 0.01, *n* = 28 observations, Wilcoxon Signed Rank Test, Two-sided).

These results reveal a tendency for clinicians in the control conditions to increase the acuity (i.e. “urgency”) of care for all patients as a result of independent reflection, leading to an increase in overtreatment. By contrast, in the network conditions, clinicians adjusted their recommendations toward safer, more equitable care for both patients, significantly reducing both unsafe undertreatment (Option A) and overtreatment (Option D). Additional sensitivity analyses show these findings to be robust to variations in clinicians’ characteristics^26^ (see SI, “Sensitivity Analyses”).

## Discussion

Past experimental and epidemiologic studies of bias have reported changes in biased attitudes as a result of cognitive interventions (such as cultural competency training)^27,28^. However, these studies have been unable to demonstrate any effect of these interventions on clinical recommendations, or on the reduction of population level disparities in clinical treatment by race and gender^6,29^.

We found that among a population of clinicians who initially exhibited significant bias in the provision of recommended treatment for a Black female versus a white male patient with chest pain, egalitarian communication networks significantly reduced disparities in treatment recommendations for the white male and Black female patient. In particular, as a result of information exchange in structured peer networks, significantly fewer clinicians recommended unsafe undertreatment for the Black female patient. Consistent with our predictions about the effects of peer-network communications in reducing biased perceptions^12,13,15,16^, these findings suggest that clinical decision-making can be viewed through a behavioral and social lens rather than as a purely individual, rational process^30^. New institutional opportunities may exist for digital technologies to connect clinicians in uniform information-sharing networks, particularly in the emerging fields of telemedicine and online clinical support networks^12,31–35^.

Our study design offered several advantages. First, by using identically clothed standardized patients, the same examination room backdrop, the same electrocardiogram exhibit, identical hand gestures and body language, and a single script, we minimized the effects of individual patient differences, for example in perceived socioeconomic status, as well as other incidental factors like patient affect, from our experiment (see SI, “Stimuli Design”). The only variation between patient conditions was the race and gender of the patient. These controls enabled the identification of bias in clinicians’ recommendations. Second, clinicians in our study were only compensated based on the quality of their final recommendations. This design created strong incentives for clinicians to provide the highest quality care. Finally, the use of several rounds of independent reflection in the control conditions ensured that any improvements in the quality of clinicians’ recommendations in the network conditions can be attributed directly to peer networks and not to the opportunity for reflection.

As with all experimental settings, the controlled design of our study necessarily comes with some limitations. First, rather than in-person clinical visits, we used video recordings of actors portraying patients and a computerized survey instrument to assess clinical treatment recommendations for the management of cardiac chest pain. This enabled us to better identify patient race and gender as the primary factors differing across patient conditions. Previous studies have demonstrated the external validity of case vignettes for assessing in-person clinical decision-making and treatment recommendations^33,35^. Further work has also indicated that using standardized patient videos, rather than written vignettes, substantially increases the likelihood that observed clinician decision-making in the study will match clinical decision-making in real medical settings^36^. Second, a practical limitation of our study, which arose due to the studio time required to hire actors and record very similar patient videos for both patients (in terms of clothing, posture, gesticulations, and pacing of speech), was that we were only able to have a single patient video for each condition. We note that a larger number of patient videos would be desirable for future studies, and we anticipate that future work will explore the extent to which additional factors may be relevant for understanding the impact of patients’ non-medical characteristics on the quality of their medical treatment. To provide support for future studies we have made the media resources constructed for this study publicly available for use by other scholars. A third limitation of our study is that we recruited participants through social media and through an academic medical center. Clinicians who responded to our invitation were likely to be younger, and more likely to be located in an academic practice, than the overall population of practicing clinicians in the US^37,38^. This suggests that the baseline bias detected in this study may be different in other populations. We also anticipate that the increasing familiarity with social technologies found among early career clinicians will be a positive factor for considering opportunities for the use of information-exchange networks to support bias reduction^38^. Finally, clinicians in our study were forced to select among four possible treatment options. These four options did not reflect the full breadth of potential clinical care options. However, the clinical options used in our study were sufficient to distinguish between recommendations for unsafe care and guideline-recommended care, revealing inequity in treatment recommendations according to the race and gender of the patient.

We found that independent reflection in the control conditions produced a consistent movement toward increased acuity treatments^39,40^. Strikingly, this movement did not have any significant effect on reducing inequity, but did significantly increase overtreatment. Peer networks may also be an effective approach to address overtreatment, an area of increasing concern in healthcare, and a well-known issue for cardiac catheterization^41–45^. Overtreatment may result in inappropriate care which not only has implications for patient outcomes but also for healthcare costs. By potentially reducing over-testing and enabling clinicians to reach a guideline-recommended treatment decision more quickly, peer networks may have the potential to reduce costs associated with diagnostic delays, inappropriate testing, and incorrect treatment^41,42^. While more work is needed to explore the economic implications of peer-network technologies for supporting clinical decisions, our findings suggest that there may be significant economic benefits of leveraging peer-network strategies to reduce both medical bias and patient mistreatment.

We anticipate that, beyond cardiovascular disease, structured peer communication networks may also be effective for reducing bias in other clinical settings known to suffer from race and gender disparities, such as the use of opioids in the management of acute pain^5,46^, imaging for back pain^47^ and breast cancer^48^, and the management of depression^49^. Our findings suggest that bias in healthcare might be treated not only as a cognitive problem, but also as a problem of social norms, which may be addressed through peer networking strategies for bias reduction.

## Methods

This research was approved by the Institutional Review Board at the University of Pennsylvania where this study was conducted, and it included informed consent by all participants in the study.

### Debriefing materials

Immediately following completion of the study, all participants were provided debriefing materials that included the correct diagnostic estimate, the correct treatment recommendation, and a detailed explanation of the clinical case, along with supporting references. The debriefing text is as follows:

“For the risk estimate, the correct answer is: 16% chance of an adverse cardiac event within 30 days. For the treatment recommendation, the correct answer is: Option C: Full-dose aspirin and refer to the emergency department for evaluation and monitoring.

#### Explanation of the answer

The patient is at intermediate/moderate risk due to: (1) symptoms (discomfort with exertion, dyspnea), (2) history (concern for cardiac origin), (3) age (>65 years old), (4) EKG (T-wave inversion / flattening), (5) risk factors (hyperlipidemia). The patient has a HEART score of 5 (1 point for moderately suspicious history; 1 point for repolarization disturbance; 2 points for age >65; 1 point for 1–2 risk factors) without a troponin level. For a HEART score range from 4 to 6, the most accurate answer is 16% chance of an adverse cardiac event within 30 days. Even a mild troponin increase would place the patient at 7 points (or high risk). The recommendation for this patient who also has T-wave abnormalities is for same day troponin testing or further evaluation in the emergency department. The patient needs to be immediately evaluated for further risk stratification via cardiac enzymes or a same day non-invasive stress testing, and therefore option C is the preferred answer. Option A does not pursue necessary further evaluation. Option B delays this evaluation. Option D is not appropriate for an individual with intermediate risk.

#### Citations

Bosner, S. et al. Ruling out coronary artery disease in primary care: development and validation of a simple prediction rule. *Can. Med. Assoc. J*. **182**, 1295–1300 (2010).

Ebell, M. H. Evaluation of chest pain in primary care patients. *Am. Fam. Physician*. **83**, 603–605 (2011).

Mahler, S. A. et al. The HEART pathway randomized trial. *Circulation* **8**(2), 195–203 (2015).

Poldervaart, J. M. et al. Effect of using the HEART Score in patients with chest pain in the Emergency Department: a stepped-wedge, cluster randomized trial. *Ann. Intern. Med*. **166**, 689–697 (2017).

### Recruitment

A total of 840 clinicians were recruited from around the US to participate in a diagnostic challenge facilitated by a mobile application designed for this study called “DxChallenge.” Clinicians were recruited between March 1 and November 29, 2019 from online discussion boards on Reddit and from Facebook’s advertising platforms, as well as through Penn Medicine’s Graduate Medical Education training program (for resident MD clinicians). Each advertisement directed clinicians to a webpage that specified the purpose of the research, the eligibility requirements, and the research compensation to interested participants. The webpage provided links to Google Play or the Apple App store, where participants could enroll by downloading the “DxChallenge” app for free. When registering in the app, participants were required to input a valid email address and a valid 10-digit National Provider Identification (NPI), i.e. the unique personal identifier given to healthcare providers in the US. The webpage informed clinicians that each diagnostic challenge would be announced via push notifications on their phone, which would appear on their screen and could be clicked to take them into the trial. The “DxChallenge” app was developed by the authors solely for the purpose of conducting this study, and the use of the DxChallenge app for this research is compliant with the terms of use for this app.

### Experimental design

To initiate a trial, the DxChallenge app sent push notifications to all clinicians who had registered for the study (Supplementary Fig. 3). Once 120 clinicians had responded, they were randomized to conditions in a 2:1 ratio—80 clinicians were randomized to the network conditions, and 40 clinicians were randomized to the control conditions (Supplementary Fig-. 1). The 80 clinicians randomized to the network condition were then randomized in a 1:1 ratio into each of the network conditions (i.e. a standardized patient video of a white male patient-actor, or a standardize patient video of a Black female patient-actor). The 40 clinicians in the control condition were then randomized in a 1:1 ratio into each of the control conditions (i.e. a standardized patient video of a white male patient-actor, or a standardize patient video of a Black female patient-actor). All randomizations were automated through the app.

In the control conditions, clinicians were isolated and not embedded in social networks. In the network conditions, clinicians were randomly assigned to a single location in a large uniform social network (*n* = 40), in which every clinician had four anonymous network contacts (Supplementary Fig. 4). Each network of 40 formed an interconnected chain of clinicians, each of whom had four direct contacts. Clinicians’ contacts in the network remained the same throughout the experiment. This created a structurally uniform network, defined as a topology in which every clinician had an equal number of connections (*z* = 4), which ensured that no single clinician had greater power over the communication dynamics within the network^13,16^. More technically, for the network condition, we generated a random k-regular graph in which every node possessed exactly four connections; to generate this graph randomly, we first generated a k-regular lattice (*k* = 4), and then we randomly rewired each connection, while making sure that every node retained only four connections. Clinicians in the network condition were then randomly assigned to a position within this randomly generated egalitarian network. The same network topology was used across all trials in the network condition.

Each clinician viewed a standardized patient video of either a white male patient-actor or Black female patient-actor, and provided clinical assessments and treatment recommendations for the depicted clinical case (see Supplementary Fig. 5 and Supplementary Fig. 6 below). Both the white male and Black female “patients” in the videos were portrayed by professional actors who appeared 65 years old, were dressed in identical attire, and depicted a patient with clinically significant chest pain symptoms. The patient-actors were recruited through a local casting service company (Kathy Wickline Casting) located in Philadelphia. An initial pool of 20 actors’ resumes and photos were reviewed by two researchers from the team. Two Black female and four white male actors were invited for sending in a test video where they narrated the female or the male patient script. All researchers reviewed the test videos, discussed their acting qualities and comparability in patient characteristics, and reached a consensus on selecting one Black female and one white male actor for the experiment. The two actors came to the media production studio of the Annenberg School for Communication on February 27, 2019. They were given the same clothes and light patient make-ups for quality comparison. All videos were filmed by the professional filming crew on the same day at the studio. Hereafter, we refer to the patient-actors in the standardized patient videos as “patients”.

In all four conditions, clinicians were asked to provide an initial evaluation of the patient video. All clinicians initially independently viewed the video and were then given two minutes to provide responses to the assessment and recommendation questions. All conditions viewed the same clinical vignette (see SI for full description of the vignette; Supplementary Figs. 5–7). Every aspect of the vignette was held constant across conditions, except for the race and gender of the patient in the video vignette. Regardless of the patient’s demographic, the patient wore the same clothing in the same environment, and the patient reported their symptoms using the same script. (See “Stimuli Design” for comprehensive detail on the structure of the vignette). All stimuli are publicly available for use in future research at the following link: https://github.com/drguilbe/cliniciansCI.

The vignette was displayed in the app. The patient’s symptoms were communicated by the patient-actor in an embedded video within the app (Supplementary Fig. 5). Each round, clinicians were given a question concerning the medical status of a patient and were asked to enter a diagnostic assessment in the “provide estimate” field. The “Clinical Recommendation” field provided a dropdown menu from which clinicians selected a clinical recommendation for the patient in the vignette. The case description for each vignette was designed in consultation with clinicians to represent the type of question that clinicians regularly face in board exams or continuing medical education exams, where the question has a preferred answer for both the probability of the specific condition and the proper clinical recommendation for patient management.

In round one, each clinician was asked to input a diagnostic assessment and a choice of treatment from a set of options in a dropdown menu (Supplementary Fig. 5). In round two and round three in the control condition, clinicians were shown the same vignette and were asked to answer the same question on their own, with no change to the user experience (Supplementary Fig. 6). In round two and round three in the network condition, clinicians were shown the average answer of the clinicians they were connected to in the social network structured through the DxChallenge app, and they were once again asked to provide a diagnostic assessment and to select a treatment option (Supplementary Fig. 6). The participant experience was identical between the control and the network condition, except for that participants in the network condition were exposed to the average assessment of the other clinicians they were connected to in the network. If at any point a participant attempted to advance to the next round without inputting a diagnostic assessment or a treatment choice, a message appeared telling them that they had to input all required responses before advancing. Each trial lasted for 8 min. Only clinicians who provided the guideline-recommended clinical recommendation in their final response were given a financial reward of $30. Clinicians who provided incorrect responses were not compensated for their participation.

### Reporting summary

Further information on research design is available in the Nature Research Reporting Summary linked to this article.

## Supplementary information

Supplementary Information

Reporting summary

## Data Availability

The data collected for this study are available for download from the Network Dynamics Group website: https://ndg.asc.upenn.edu/experiments/physician-reasoning/. The data are also available at https://github.com/drguilbe/cliniciansCI. Source data are provided with this paper.

## Source data

Source Data

## Code availability

The code for this study was written in R, and is deposited on GitHub, available at: https://github.com/drguilbe/cliniciansCI.
