# Supplementary material for: The reduction of race and gender bias in clinical treatment recommendations using clinician peer networks in an experimental setting
Source: Nat Commun. 2021 Nov 15;12:6585. doi: 10.1038/s41467-021-26905-5 (PMC8593068; doi:10.1038/s41467-021-26905-5)
Supplement: Supplementary file 1 — Supplementary Information [file 41467_2021_26905_MOESM1_ESM.pdf]

Supplementary Information for:

The reduction of race and gender bias in clinical treatment recommendations using clinician peer networks in an experimental setting

This file includes:

Supplementary Methods
Supplementary Discussion
Supplementary References

Supplementary Methods

Subject Recruitment and Demographics

Clinicians were recruited from around the US by distributing advertisements over clinician discussion boards on Reddit and Facebook's advertising platforms. Seven recruitment advertisements were posted on Reddit, specifically on messaging boards that attract doctors and resident clinicians. We distributed three advertisements over Facebook, from March to November 2019, while making use of Facebook's advertising platform to target clinicians. We limited advertisement exposure to people who resided in the US, who were 18 to 65, and whose demographic characteristics were among the following features suggested by Facebook: doctor (Dr), medical doctor (MD), and medical director (MD). Beyond online recruitment, clinicians were also recruited through Penn Medicine's Graduate Medical Education training program (for resident MD clinicians). Advertisements were circulated to the 2017 cohort of resident clinicians, and clinicians were also recruited through outreach events as part of Penn Medicine's orientation for incoming residents.

Each advertisement directed clinicians to a webpage that specified the purpose of the research, eligibility requirement, and research compensation to interested participants. The webpage provided links to Google Play or the Apple App store, where participants could enroll by downloading the proprietary app called "DxChallenge" for free. The webpage informed clinicians that each diagnostic challenge would be announced via push notifications on their phone, which would appear on their screen and could be clicked to take them into the trial. The mobile application "DxChallenge" was developed by the authors solely for the purpose of conducting this study, and the use of the DxChallenge app for this research is compliant with the terms of use for this app.

When registering in the app, participants were required to input a valid email address and a valid 10-digit National Provider Identification (NPI), i.e., the unique personal identifier given to health care providers in the US. Each NPI could be queried in a public registry to obtain the state in which a given clinician was registered to practice as a health care provider.

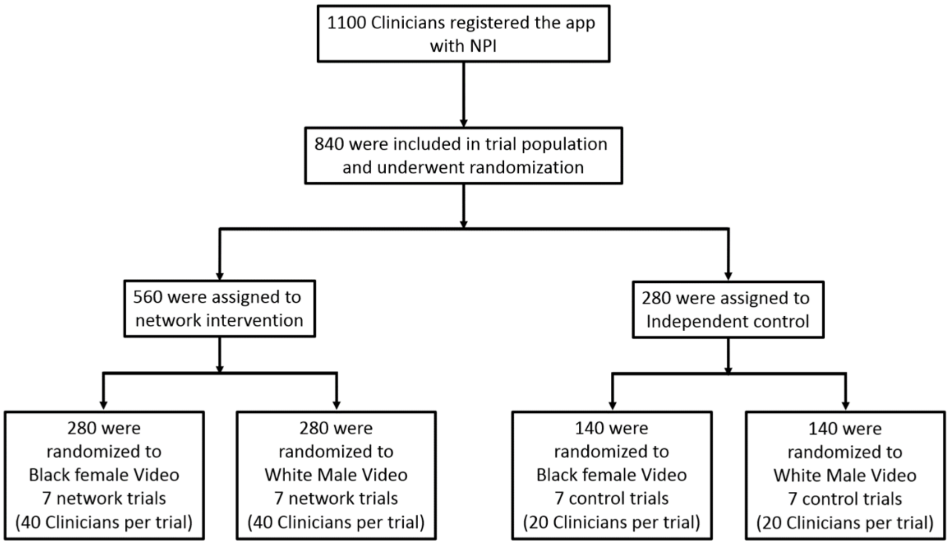

Supplementary Figure 1: **Enrollment, Intention-to-Treat Sample Size, and Randomization of Clinicians.** Fewer clinicians were randomized to the control conditions on each trial because the statistical independence of clinicians in these conditions provided

greater statistical power and enabled bootstrapping techniques to generate paired trial-level comparisons (see “Statistical Analyses”).

From March 1, 2019 to November 29, 2019, we recruited 1100 clinicians of whom 840 responded (560 network, 260 control) to one of the push notifications for this study (Supplementary Figure 1). Baseline characteristics did not differ significantly between the two groups except for the date of NPI assignment, with more clinicians with NPI assignments in 2009-2012 assigned to the control condition (Supplementary Table 1). Supplementary Figure 2 displays the geographical location of the clinicians that made up the recruitment pool for this study.

	**Group: *n* (%)**	
**Trait**	Intervention (network)<br>*n*=560	Control (independent)<br>*n*=280
**Gender**		
Male	62.4%	64.8%
Female	37.5%	35.1%
**Date of NPI assignment**		
2017 -	49.8%	45.4%
2013 – 2016	32.8%	32.2%
2009 – 2012	7.0%	13.6%
2005 – 2008	10.3%	8.6%
**Primary Care**	91.6%	87.0%
**Independent practice**	23.4%	21.9%

Supplementary Table 1: **Participating clinician demographic traits**.

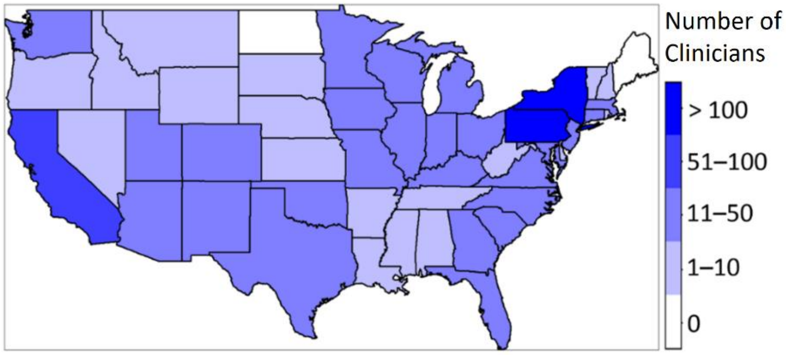

Supplementary Figure 2: **Displayed is the geographic distribution of clinicians from across the US who were recruited for this study using the**

> **DxChallenge app.** The geographic distribution of our participant pool was determined using the NPI ('National Provider Number') of each clinician, which they were required to input when registering in the app. The NPI for each clinician indicates the state in which they gained their license to practice as a health care provider.

Trial Design

To initiate a trial, the app sent push notifications to all 1100 clinicians who had registered for the study (Supplementary Figure 3). Once 120 clinicians had responded, they were randomized to conditions in a 2:1 ratio – 80 clinicians were randomized to the network conditions, and 40 clinicians were randomized to the control conditions (Supplementary Figure 1). The 80 clinicians randomized to the network condition were then randomized in a 1:1 ratio into each of the network conditions (i.e., a standardized patient video of a white male patient-actor, or a standardize patient video of a Black female patient-actor). The 40 clinicians in the control condition were then randomized in a 1:1 ratio into each of the control conditions (i.e., a standardized patient video of a white male patient-actor, or a standardize patient video of a Black female patient-actor). All randomizations were automated through the app. (See "Statistical Analyses" for greater detail).

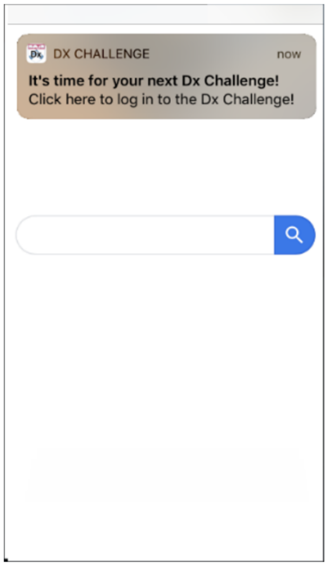

Supplementary Figure 3: **Displayed is a screenshot of the push notification clinicians received to invite them to participate in a diagnostic challenge.**

In the control conditions, clinicians were isolated and not embedded in social networks. In the network conditions, clinicians were randomly assigned to a single location in a large uniform social network (n=40), in which every clinician had four anonymous network contacts (Supplementary Figure 4). Each network of 40 formed an interconnected chain of clinicians, each of whom had four direct contacts. Clinicians' contacts in the network remained the same throughout the experiment. This created a structurally uniform network, defined as a topology in which every clinician had an equal number of connections (z=4), which ensured that no single clinician had greater power over the communication dynamics within the network (*1-4*). More technically, for the network condition, we generated a random k-regular graph in which every node possessed exactly 4 connections; to generate this graph randomly, we first generated a k-regular lattice (k=4), and then we randomly rewired each connection, while making sure that every node retained only 4 connections (*5*). Clinicians in the network

condition were then randomly assigned to a position within this randomly generated egalitarian network. The same network topology was used across all trials in the network condition.

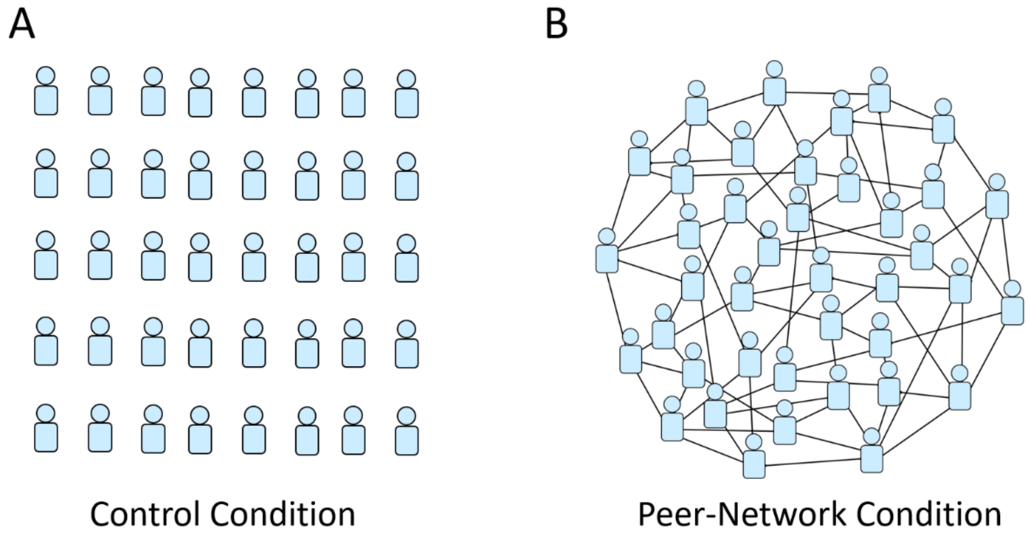

Supplementary Figure 4: A schematic display of the population structures that characterized the control (independent) condition and the peer-network condition.

Each clinician viewed a standardized patient video of either a white male patient-actor or Black female patient-actor, and provided clinical assessments and treatment recommendations for the depicted clinical case (see Supplementary Figure 5 and Supplementary Figure 6 below). Both the white male and Black female "patients" in the videos were portrayed by professional actors who appeared 65 years old, were dressed in identical attire, and depicted a patient with clinically significant chest pain symptoms. The patient-actors were recruited through a local casting service company (Kathy Wickline Casting) located in Philadelphia. An initial pool of 20 actors' resumes and photos were reviewed by two researchers from the team. Two Black female and four white male actors were invited for sending in a test video where they narrated the female or the male patient script. All researchers reviewed the test videos, discussed their

acting qualities and comparability in patient characteristics, and reached a consensus on selecting one Black female and one white male actor for the experiment. The two actors came to the media production studio of the Annenberg School for Communication on February 27 2019. They were given the same clothes and light patient make-ups for quality comparison. All videos were filmed by the professional filming crew on the same day at the studio. Hereafter, we refer to the patient-actors in the standardized patient videos as “patients”.

In all four conditions, clinicians were asked to provide an initial evaluation of the patient video. All clinicians initially independently viewed the video and were then given two minutes to provide responses to the assessment and recommendation questions. All conditions viewed the same clinical vignette. Every aspect of the vignette was held constant across conditions, except for the race and gender of the patient in the video vignette. Regardless of the patient’s demographic, the patient wore the same clothing in the same environment, and the patient reported their symptoms using the same script. (See “Stimuli Design” for comprehensive detail on the structure of the vignette). All stimuli are publicly available for use in future research at the following link: https://github.com/drguilbe/cliniciansCI.

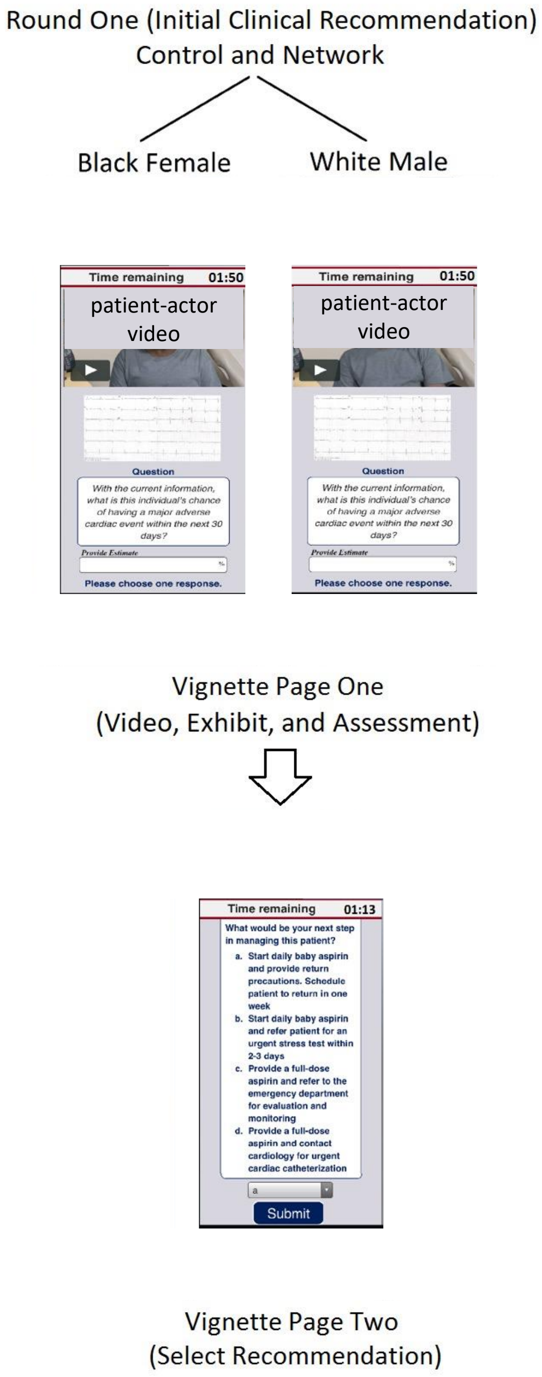

Supplementary Figure 5: **Round one for all conditions**. Image of the app and vignette at the initial clinical recommendation. Top row displays page one of the vignettes, which is held constant for conditions with the Black female and white male patients. Bottom row displays page two of the vignette (reached by scrolling downward on the screen), which was also held constant across all conditions.

The vignette was displayed in the app. The patient's symptoms were communicated by the patient-actor in an embedded video within the app (Supplementary Figure 5). Each round, clinicians were given a question concerning the medical status of a patient and were asked to enter a diagnostic assessment in the "provide estimate" field. The "Clinical Recommendation" field provided a drop-down menu from which clinicians selected a clinical recommendation for the patient in the vignette. The case description for each vignette was designed in consultation with clinicians to represent the type of question that clinicians regularly face in board exams or continuing medical education exams, where the question has a preferred answer for both the probability of the specific condition and the proper clinical recommendation for patient management.

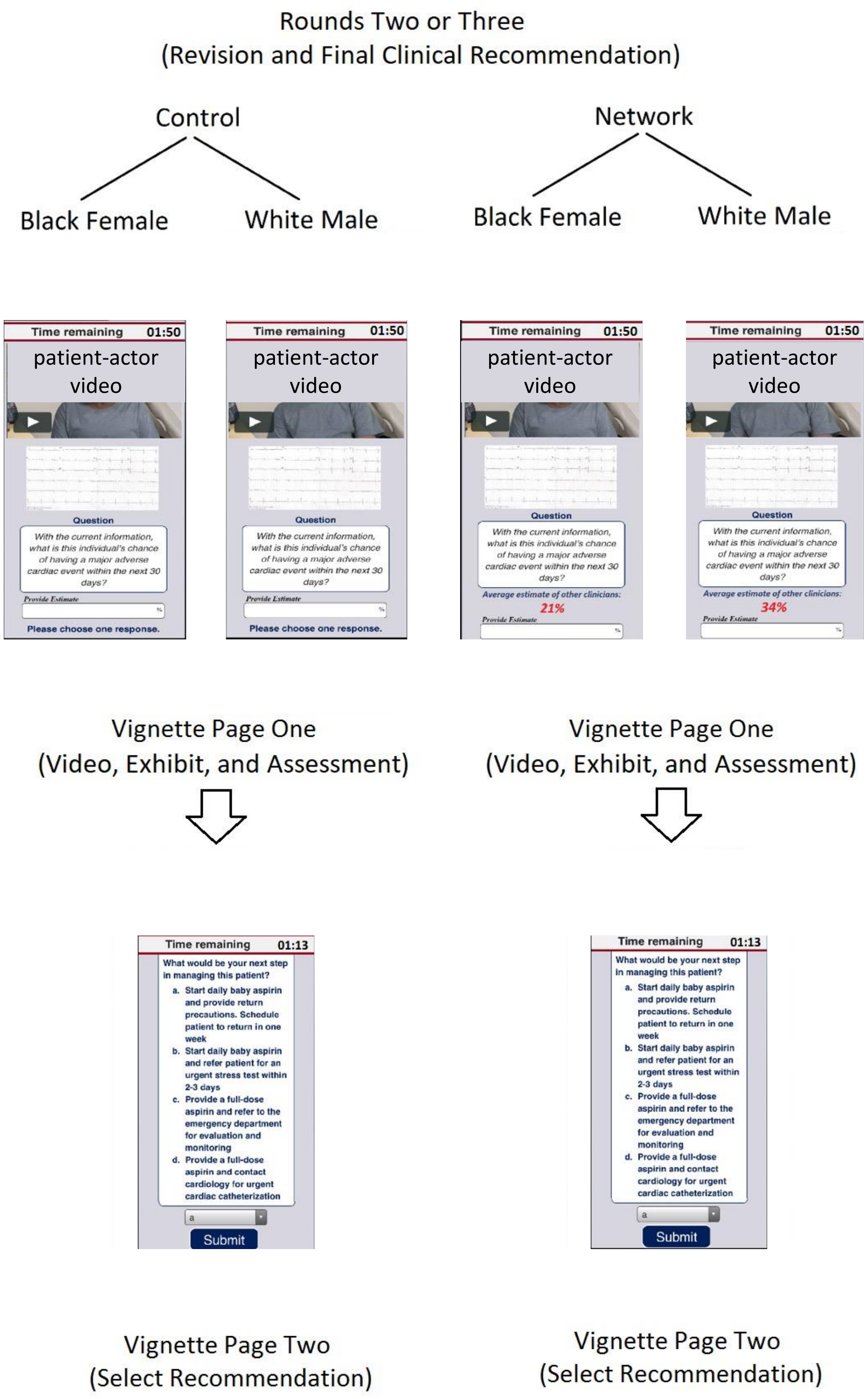

Supplementary Figure 6: **Rounds two and three for all conditions.** Image of the app and vignette at the second clinical recommendation. Top row displays page one of the

vignettes. In the control conditions, page one for the second recommendation is the same as the page for the initial recommendation. In the network conditions, page one for the second recommendation varies from page one for the initial recommendation because it displays the average assessment of clinicians' network peers. Bottom row displays page two of the vignette (reached by scrolling downward on the screen), which was held constant across all conditions.

In round one, each clinician was asked to input a diagnostic assessment and a choice of treatment from a set of options in a dropdown menu (Supplementary Figure 5). In round two and round three in the control condition, clinicians were shown the same vignette and were asked to answer the same question on their own, with no change to the user experience (Supplementary Figure 6). In round two and round three in the network condition, clinicians were shown the average answer of the clinicians they were connected to in the social network structured through the DxChallenge app, and they were once again asked to provide a diagnostic assessment and to select a treatment option (Supplementary Figure 6). The participant experience was identical between the control and the network condition, except for that participants in the network condition were exposed to the average assessment of the other clinicians they were connected to in the network. If at any point a participant attempted to advance to the next round without inputting a diagnostic assessment or a treatment choice, a message appeared telling them that they had to input all required responses before advancing.

Each trial lasted for 8 minutes. Only clinicians who provided the guideline-recommended clinical recommendation in their final response were given a financial reward of $30. Clinicians who provided incorrect responses were not compensated for their participation. These study procedures were approved by the Institutional Review Board at the University of Pennsylvania (Protocol 834377). Immediately following completion of the study, all participants were provided debriefing materials that included the correct diagnostic estimate,

the correct treatment recommendation, and a detailed explanation of the clinical case, along with supporting references. The debriefing text is as follows:

"For the risk estimate, the correct answer is: 16% chance of an adverse cardiac event within 30 days. For the treatment recommendation, the correct answer is: Option C: Full-dose aspirin and refer to the emergency department for evaluation and monitoring.

Explanation of the answer:
The patient is at intermediate/moderate risk due to: (1) symptoms (discomfort with exertion, dyspnea), 2 history (concern for cardiac origin), (3) age (>65 years old), (4) EKG (T-wave inversion / flattening), (5) risk factors (hyperlipidemia). The patient has a HEART score of 5 (1 point for moderately suspicious history; 1 point for repolarization disturbance; 2 points for age >65; 1 point for 1-2 risk factors) without a troponin level. For a HEART score range from 4-6, the most accurate answer is **16**% chance of an adverse cardiac event within 30 days. Even a mild troponin increase would place the patient at 7 points (or high risk). The recommendation for this patient who also has T-wave abnormalities is for same day troponin testing or further evaluation in the emergency department. The patient needs to be immediately evaluated for further risk stratification via cardiac enzymes or a same day noninvasive stress testing, and therefore option C is the preferred answer. Option A does not pursue necessary further evaluation. Option B delays this evaluation. Option D is not appropriate for an individual with intermediate risk.

Citations:
Bosner S, Haasenritter J, Becker A, Karatolios K, Vaucher P, Gencer B, et al. Ruling out coronary artery disease in primary care: development and validation of a simple prediction rule. Can Med Assoc J. 2010;182:1295–300.

Ebell MH. Evaluation of chest pain in primary care patients. Am Fam Physician. 2011;83:603–5.

Mahler, S. A., Riley, R. F., Hiestand, B. C., Russell, G. B., Hoekstra, J. W., et al. (2015). The HEART pathway randomized trial. Circulation: Cardiovascular Quality and Outcomes, 8(2), 195-203.

Poldervaart JM, Reitsma JB, Backus BE, Koffijberg H, Veldkamp RF, ten Haaf ME, et al. Effect of Using the HEART Score in Patients With Chest Pain in the Emergency Department: A Stepped-Wedge, Cluster Randomized Trial. Ann Intern Med. 2017;166:689–697."

Stimuli Design

Here we provide the full details for the clinical vignette used as stimuli in this experiment. The context and script of the clinical vignette was held constant regardless of the race and gender of the patient. All videos were recorded in the same studio using the same

equipment with actors instructed to use the same hand motions, facial expressions and intonations. The clinical vignette was accompanied by an exhibit of the electrocardiogram associated with the patient (Supplementary Figure 7).

Attire: Normal
Grooming: Well-groomed
Demeanor: Normal
Set / props: Sitting on exam room table

Patient Script*:*

"I'm so glad you were able to see me this afternoon. Ever since I retired a few years ago at 65, I've had time to try to get healthier. I know I'm overweight, so I've started to exercise more. After my walk this morning, I noticed a weird, tired feeling that made me feel a little short of breath. I sat down in my kitchen to get a sip of water and rest; it felt better a few minutes afterwards. I also felt fine when I walked up the stairs to your office. The medical assistant who took my vital signs said everything looks great, and I've been taking the cholesterol medication every day. So, I don't think it's a big deal, but I want to make sure since my dad had a heart attack in his early 60's.

Exhibit*:*

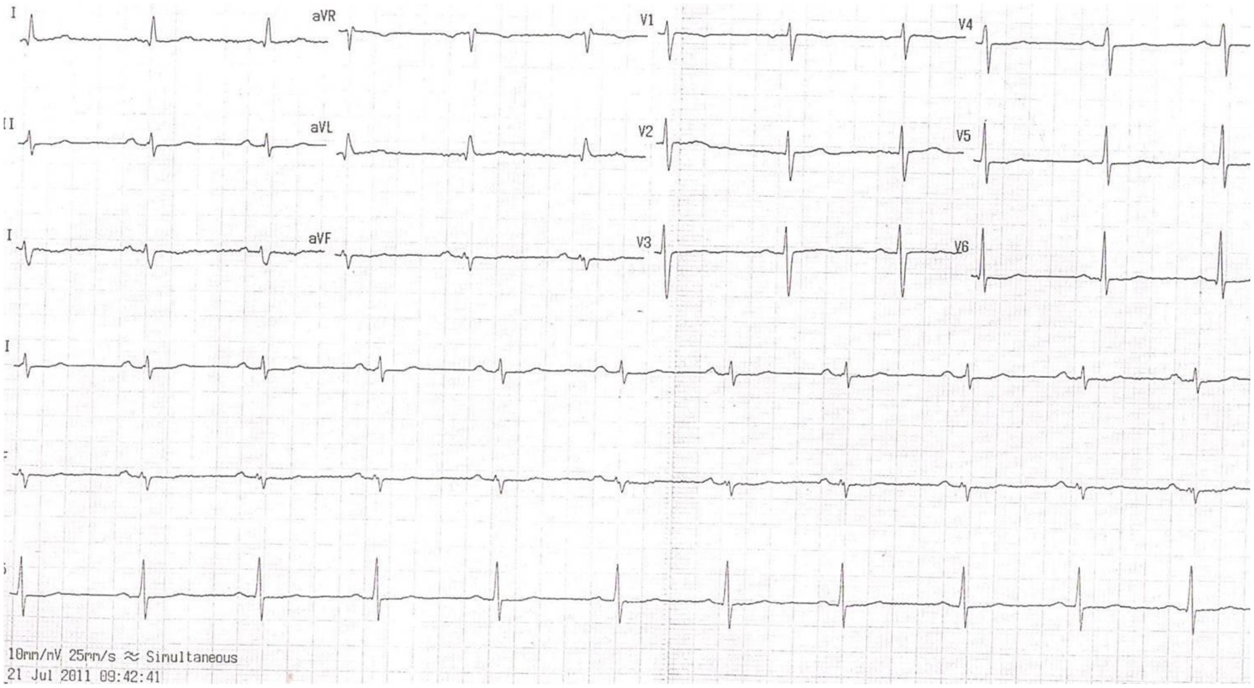

Supplementary Figure 7: Electrocardiogram exhibit provided to all participants.

Question 1:

With the current information, what is this individual's chance of having a major adverse cardiac event within the next 30 days?

The patient has a HEART score of 5 (1 point for moderately suspicious history; 1 point for repolarization disturbance; 2 points for age >65; 1 point for 1-2 risk factors) without a troponin level (*6-8*). For a HEART score range from 4-6, the most accurate answer is: **16**% chance of an adverse cardiac event within 30 days (*6,7*).

Question 2:

What would be your next step in management of this patient?

a. Start a daily baby aspirin and provide clear return precautions. Schedule the patient to return in one week.
b. Start a daily baby aspirin and refer the patient for an urgent stress test within 2-3 days.
c. Provide a full-dose aspirin and refer to the emergency department for evaluation and monitoring.
d. Provide a full-dose aspirin and contact cardiology for urgent cardiac catheterization.

The current guideline-recommended answer is **C**.

Based on several risk stratification scores, this patient is at intermediate/moderate risk due to these components (*6-10*):

- symptoms (discomfort with exertion, dyspnea)
- history (concern for cardiac origin)
- age (>65 years old)
- EKG (T-wave inversion / flattening)
- risk factors (hyperlipidemia)

In sum, the patient has a HEART score of 5 (1 point for moderately suspicious history; 1 point for repolarization disturbance; 2 points for age >65; 1 point for 1-2 risk factors) without a troponin level (*6,7*). Even a mild troponin increase would place the patient at 7 points (or high risk). Intermediate risk individuals were found to have a 16% risk of a major adverse cardiac

event (MACE) within 6 weeks and high-risk individuals had a 50-65% risk of a MACE (*6,7*). Without knowledge of a troponin level, this moderate-risk individual was further evaluated and admitted to the hospital in the HEART study (*7-10*).

A study within primary care created a risk score to determine the likelihood of coronary artery disease in patients with chest pain (*9*). From that study, the patient has a risk score of 3 (age 65+; worse during exercise; concern about cardiac origin) and potentially 4 (if pain is not reproducible by palpation). Patients with a risk score of 3+ were found to have 4.5 times likelihood of having coronary artery disease (*10*). The recommendation for this patient who also has T-wave abnormalities is for same day troponin testing or further evaluation in the emergency department (*9,10*).

Given both these studies, the patient needs to be immediately evaluated for further risk stratification via cardiac enzymes or a same day noninvasive stress testing, and therefore option C is the preferred answer. Option A does not pursue necessary further evaluation. Option B delays this evaluation. Option D is not appropriate for an individual with intermediate risk.

Statistical Analyses

Power calculations performed in 2018 determined that 7 trials (each with 120 subjects) provided the minimum bound for achieving 80% power to detect a strong anticipated effect size based on prior studies (*2-4,11*). We used the Wilcoxon Signed Rank Test, paired at the trial-level, to assess primary, secondary, and exploratory outcomes (*11*). Unless explicitly noted, all statistical analyses are implemented using trial-level analysis to control for statistical nonindependence among clinicians after they exchange diagnostic assessments in social networks. In the regression techniques we use, all standard errors are clustered at the trial level

to preserve trial-level analyses. We also undertook sensitivity analyses which ensure no significant differences in clinician traits between complete and incomplete responses, and to assess if results varied by clinician traits (see “Sensitivity Analyses”).

Because clinicians in the control condition were independent, fewer clinicians were needed in this condition to enable sufficient statistical power. For this reason, our randomization scheme assigned 40 subjects to each network condition and 20 subjects to each control condition within each trial. To facilitate paired comparisons between network and control groups within each trial, we employed bootstrapping techniques (via Hot Deck Imputation; ref. *12*) to randomly assemble 7 control groups of equivalent size (n=40 in each control group) for each control condition (*1*,*2*). To produce 7 trials of control groups in each condition, we sampled without replacement from the set of total control participants in each condition to produce independent groups of 40. Once sampling without replacement exhausted the available control pool, we initiated sampling with replacement. The result was the formation of 7 randomly organized control groups that minimized repeated sampling. This approach was viable in the control groups because clinicians in this condition were independent, such that randomly grouping them together had no impact on their within-subject changes throughout the experiment.

We defined the diagnostic accuracy of assessments as the absolute number of percentage points between a clinician’s diagnostic assessment and the most accurate diagnostic assessment. For example, given that the most accurate diagnostic assessment is 16%, a clinician who provided a diagnostic assessment of 22% would be associated with a diagnostic error of 6 percentage points; note, since this measure of error is absolute, a clinician who provided a diagnostic assessment of 10% would be associated with the same diagnostic error

of 6 percentage points. For clarity of presentation, we normalize diagnostic accuracy on a 0 to 1 scale by applying min-max normalization to the absolute error of clinicians' diagnostic assessments. Min-max normalization is defined by supplementary equation 1 below (where x_i is the diagnostic error of the individual clinician *i*; min(x) is the minimum diagnostic error across all clinicians; max(x) is the maximum diagnostic error across all conditions; and z_i is the normalized diagnostic error of the individual clinician *i*). All instances of diagnostic error in this equation are multiplied by -1 prior to normalization, so that minimum values of error correspond to maximum values of accuracy, and maximum values of error correspond to minimum values of accuracy.

$$z_i = \frac{x_i - \min(x)}{\max(x) - \min(x)} \quad (1)$$

Under this procedure, the minimum possible accuracy (indicated by 0) corresponds to the diagnostic assessment with the greatest absolute error (i.e., an estimate that is as far as possible from the most accurate answer of 16%, which in this case is 84 percentage points), while the maximum possible accuracy (indicated by 1) corresponds to a diagnostic assessment that is 0 percentage points away from the most accurate answer (such that they are equivalent). A key advantage of this normalization technique is that a single percentage point change in the outcome variable continues to be equivalent to a single percentage point change in the absolute error of a diagnostic assessment; in other words, a 3 percentage point reduction in normalized diagnostic error corresponds to a 3 percentage point reduction in absolute error.

To measure inequity, we adopt a difference in difference approach, akin to popular measures of polarization (*1*,*2*). This difference in difference approach is calculated at the trial level. For instance, on a given trial, we calculate inequity for round 1 by (i) in each condition,

taking the difference between the percent of clinicians who recommended option A (unsafe undertreatment) and the percent of clinicians who recommended option C (guideline-recommended treatment) for the Black female patient; then (ii) in each condition, taking the difference between the percent of clinicians who recommended option A (unsafe undertreatment) and the percent of clinicians who recommended option C (guideline-recommended treatment) for the white male patient; and finally (iii), taking the difference between the differences calculated for (i) the Black female patient and (ii) the white male patient. We undertook this procedure for each trial, producing 7 trial-level measures of inequity for each experimental condition and for each round. This measure of inequity is represented with the following equation:

$$I_{\mathrm{T,C,R}} = (A\% - C\%)_{BF} - (A\% - C\%)_{WM} \quad (2)$$

In supplementary equation (2), "I" refers to inequity; "T" refers to the trial used for the calculation; "C" refers to the experimental condition (either Control or Network); "R" refers to the round of recommendation (Initial, Second, or Final) used for the calculation; "A%" refers to the percent of clinicians selecting option A (unsafe undertreatment) on round R for condition C in trial T; "C%" refers to the percent of clinicians selecting option C (guideline-recommended treatment) on round R for condition C in trial T; "BF" refers to the trial data corresponding to the Black female patient; and "WM" refers to the trial data corresponding to the white male patient. On a given trial on a given round, if inequity is positive, this indicates that clinicians are more likely to recommend unsafe undertreatment vs. guideline-recommended treatment, A vs. C, to the Black female patient rather than the white male patient.

For a comprehensive overview of our main results, we provide a series of tables which present the raw percent of clinicians in each round of each trial in each condition who selected each treatment option. Supplementary Table 2 presents this data for the control condition. Supplementary Table 3 presents this data for the network condition. Supplementary Table 4 presents the same data, averaged across all trials in the control condition. Supplementary Table 5 presents the same averaged across all trials in the network condition.

		Control Condition			
		Initial		Final	
Trial	Recommendation	Black female	white male	Black female	white male
1	A	39%	21%	24%	10%
1	B	52%	50%	51%	50%
1	C	9%	29%	24%	30%
1	D	0%	0%	0%	10%
2	A	27%	30%	18%	32%
2	B	61%	39%	57%	41%
2	C	12%	24%	15%	19%
2	D	0%	6%	10%	8%
3	A	23%	14%	40%	22%
3	B	55%	58%	43%	44%
3	C	16%	19%	13%	31%
3	D	6%	8%	3%	3%
4	A	41%	21%	21%	12%
4	B	47%	55%	47%	48%
4	C	12%	24%	32%	28%
4	D	0%	0%	0%	12%
5	A	26%	29%	27%	28%
5	B	66%	37%	57%	39%
5	C	9%	29%	5%	28%
5	D	0%	6%	11%	6%
6	A	20%	21%	34%	27%
6	B	53%	59%	50%	52%
6	C	17%	15%	9%	18%
6	D	10%	6%	6%	3%
7	A	29%	9%	20%	9%

7	B	52%	59%	54%	59%
7	C	19%	32%	17%	26%
7	D	0%	0%	9%	6%

Supplementary Table 2: The percent of clinicians in each round of each trial in the control condition who selected each of the possible treatment recommendations.

		Network Condition<br>Initial		Final	
Trial	Recommendation	Black female	white male	Black female	white male
1	A	35%	27%	11%	12%
1	B	50%	55%	51%	62%
1	C	4%	18%	34%	27%
1	D	12%	0%	3%	0%
2	A	29%	30%	9%	17%
2	B	48%	48%	52%	62%
2	C	23%	22%	30%	21%
2	D	0%	0%	9%	0%
3	A	55%	20%	25%	21%
3	B	31%	53%	56%	50%
3	C	7%	27%	17%	24%
3	D	7%	0%	3%	6%
4	A	42%	31%	25%	21%
4	B	42%	50%	53%	68%
4	C	4%	12%	12%	11%
4	D	12%	8%	9%	0%
5	A	20%	29%	11%	32%
5	B	50%	46%	67%	32%
5	C	20%	25%	22%	32%
5	D	10%	0%	0%	5%
6	A	7%	29%	10%	14%
6	B	52%	61%	43%	66%
6	C	33%	11%	48%	21%
6	D	7%	0%	0%	0%
7	A	27%	22%	6%	9%
7	B	53%	59%	62%	57%
7	C	13%	15%	31%	30%
7	D	7%	4%	0%	4%

Supplementary Table 3: The percent of clinicians in each round of each trial in the network condition who selected each of the possible treatment recommendations.

Control Condition<br>Initial			Final	
Recommendation	Black female	white male	Black female	white male
A	29.29%	20.71%	26.29%	20%
B	55.14%	51%	51.29%	47.57%
C	13.43%	24.57%	16.43%	25.71%
D	2.29%	3.71%	5.57%	6.86%

Supplementary Table 4: **The average percent of clinicians in each round in the control condition who selected each of the possible treatment recommendations.**

Network Condition<br>Round One			Round Three	
Recommendation	Black female	white male	Black female	white male
A	30.65%	26.33%	13.91%	19.63%
B	46.61%	52.18%	54.80%	57.00%
C	14.87%	18.13%	27.83%	21.14%
D	9.1%	7.79%	6.02%	5.16%

Supplementary Table 5: **The average percent of clinicians in each round in the network condition who selected each of the possible treatment recommendations.**

Background for the Study Design

The design of this study builds on previous studies of networked collective intelligence (*1-4*), which show the reproducible dynamics of increased collective intelligence and reduced bias within egalitarian social networks. An early study (*1*) found that these improvements in collective intelligence are not due to the expected mechanism of a "regression to mean," in which everyone converges toward the average answer of the group (often referred to as the "wisdom of the crowd"). Instead, contrary to expectation, these studies found that the mean of the population distribution could improve by virtue of the network dynamics of collective intelligence. This study (*1*) identified the mechanism for this collective improvement to be the

"revision coefficient": the correlation between individual accuracy and magnitude of revision. While previous theoretical analyses had assumed that individuals' revision magnitude was identically and independently distributed (I.I.D.) across the population (i.e., a uniform random distribution), this study (*1*) showed that revision magnitude was significantly correlated with accuracy: more accurate people revised their answers less than less accurate people (referred to as a "positive revision coefficient").

A second finding (*1*) was that the revision coefficient had no effect on the collective outcome in centralized network structures (i.e., networks with highly skewed degree distributions). However, if the network was egalitarian (i.e., a uniform degree distribution) then people in the network would adjust their answers toward the responses of the more accurate people. Thus, in egalitarian networks accurate individuals became "centers of gravity" during the revision process, shifting the population mean toward the correct answer. As described in (*1*), the reason this population improvement only occurs in egalitarian networks is that the uniform structure of the network ensures that everyone has equal influence, which results in the dynamics of social influence being governed by the magnitude of revision rather than by network position: i.e., people who revise their answers less become attractors for collective changes in beliefs throughout the entire network. These results were found to hold across diverse topics (*1*), such that across multiple questions on multiple topics, the process of social learning in decentralized networks consistently produced a shift in the distribution of responses that improved the collective intelligence of the population above the traditional wisdom of the crowd (i.e., the control condition).

Subsequent studies (*2,3*) extended these findings to the reduction of partisan bias among Democrats and Republicans engaged in the evaluation of (respectively) climate change

trends (*3*) and immigration statistics (*2*). These findings were subsequently extended into the health domain with the use of egalitarian information-exchange networks to reduce bias in smokers' interpretations of smoking risks (*4*).

Supplementary Discussion

Comparing Initial Diagnostic Assessments across Conditions

Here, we report that there are no significant differences in the initial diagnostic assessments provided by clinicians in the control and the network conditions, for either patient demographic. To evaluate this comparison, Table S6 presents an OLS regression predicting the initial (i.e., Round 1) diagnostic assessments of clinicians as a function of experimental condition and patient demographic. Table S6 indicates that an interaction term between experimental condition and patient demographic is unrelated to the initial diagnostic assessment of clinicians ($\beta_{\text{[Patient Demographic * Experimental Condition]}}$=1.06, CI=[-3.79 – 5.92], p=0.66).

	**Initial Diagnostic Assessment**			
Predictors	*Estimates*	*std. Error*	*CI*	*p*
(Intercept)	17.43	1.36	14.77 – 20.09	**<0.001**
Patient Demographic [White male]	-0.06	1.86	-3.72 – 3.59	0.972
Experimental Condition [Control]	3.20	1.79	-0.31 – 6.72	0.074
Patient Demographic [White male] * Experimental Condition [Control]	1.06	2.47	-3.79 – 5.92	0.667
Observations	808			
R^2 / R^2 adjusted	0.012 / 0.008			

Supplementary Table 6: **An OLS regression predicting the initial (i.e., Round 1) diagnostic assessments of clinicians as a function of experimental condition**

and patient demographic. All statistical comparisons are two-sided. Bolded p-values indicate statistical significance at the p<0.05 level.

A complete view of the effect sizes reported in Supplementary Table 6 are visualized in Supplementary Figure 8 below, which illustrates that there are no significant differences in the initial diagnostic assessments of clinicians in the control and network condition, compared within each patient demographic (as indicated by the highly overlapping error bars).

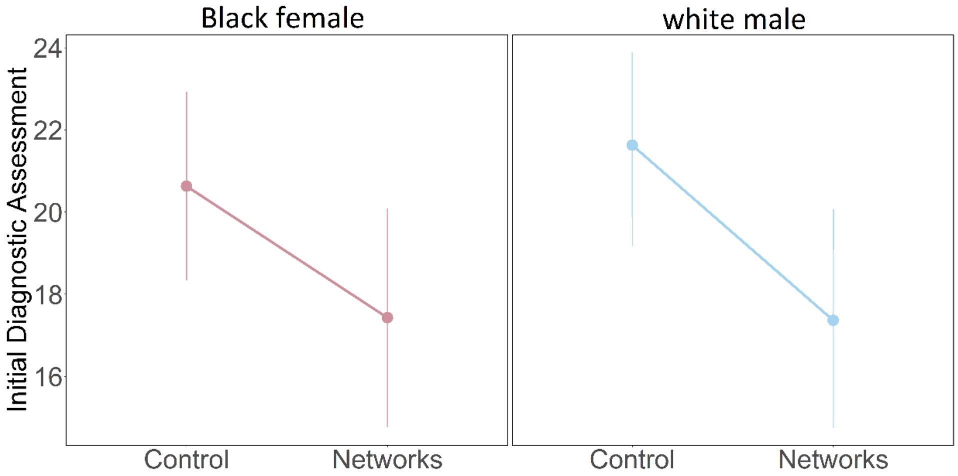

Supplementary Figure 8: **The initial diagnostic assessments of clinicians, differentiated by experimental condition and patient demographic**. Results are first averaged within each trial in each condition, and then across trials (*N*=7) in each condition. 95% confidence intervals are shown.

Individual-level Analyses

Our main results are reported at the trial-level to control for statistical nonindependence among clinicians in the network condition. This approach was adopted because, while it weakens statistical power, it is a more conservative causal test of the hypothesized effect. Here we show that our main results are consistent with – and are in fact stronger – using individual-level analyses that adjust for statistical nonindependence by clustering standard errors at the trial level for both control and network groups.

	Initial Probability of Guideline-Recommended Treatment			
Predictors	*Odds Ratios*	*std. Error*	*CI*	*p*
(Intercept)	0.10	0.21	0.07 – 0.15	**<0.001**
Initial Diagnostic Assessment	1.02	0.00	1.01 – 1.03	**<0.001**
Patient Demographic [white male]	1.78	0.19	1.22 – 2.60	**0.003**
Experimental Condition [Control]	1.05	0.19	0.72 – 1.52	0.812
Observations	808			
R^2 Tjur	0.035			

Supplementary Table 7: **Logistic regression examining baseline bias in the probability of recommending the guideline-recommended clinical treatment as a function of patient demographic, controlling for experimental condition and initial diagnostic assessment**. All statistical comparisons are two-sided. Bolded p-values indicate statistical significance at the p<0.05 level.

Supplementary Table 7 displays the results of a logistic regression including all conditions showing that clinicians were significantly more likely to provide the guideline-recommended clinical recommendation (option C) to the white male patient than the Black female patient, at the time of their initial recommendation (p=0.003, OR=1.78, n=808). Importantly, Table S7 also shows that this baseline bias is significant while controlling for experimental condition, and there was no significant difference in the level of baseline bias observed across experimental conditions (p=0.81, OR=1.05, n=808). Lastly, Table S7 further shows that the baseline bias in treatment recommendations holds even when controlling for the initial diagnostic assessments provided by clinicians, indicating that clinicians were more likely to recommend the guideline-recommended treatment to white male patients, regardless of the accuracy of their initial diagnostic assessments.

	Initial Probability of Unsafe Undertreatment			
Predictors	*Odds Ratios*	*std. Error*	*CI*	*p*
(Intercept)	0.51	0.17	0.37 – 0.71	**<0.001**
Initial Diagnostic Assessment	0.97	0.01	0.96 – 0.99	**<0.001**
Patient Demographic [Black female]	1.49	0.16	1.08 – 2.04	**0.015**
Experimental Condition [Control]	0.87	0.16	0.64 – 1.20	0.413
Observations	808			
R^2 Tjur	0.040			

Supplementary Table 8: **Logistic regression examining baseline bias in the probability of recommending unsafe undertreatment as a function of patient demographic, controlling for experimental condition and initial diagnostic assessment**. All statistical comparisons are two-sided. Bolded p-values indicate statistical significance at the p<0.05 level.

Supplementary Table 8 displays the results of a logistic regression including all conditions showing that clinicians were significantly more likely to recommend unsafe undertreatment (option A) to the Black female patient than the white male patient, at the time of their initial recommendation (p=0.01, OR=1.49, n=808). Importantly, Supplementary Table 8 also shows that this baseline bias is significant while controlling for experimental condition, and there was no significant difference in the probability of initially recommending unsafe undertreatment across experimental conditions (p=0.41, OR=0.87, n=808). As expected, Table S8 shows that lower diagnostic assessments (those corresponding to lower estimates of risk) are associated with an increased probability of recommending unsafe undertreatment. Yet, Supplementary Table 8 also shows that the baseline bias in recommending unsafe

undertreatment holds even when controlling for the initial diagnostic assessments provided by clinicians, indicating that clinicians were more likely to recommend the unsafe undertreatment to Black female patients, regardless of the accuracy of their initial diagnostic assessments.

	Probability of Improving Treatment Recommendation			
Predictors	*Odds Ratios*	*std. Error*	*CI*	*p*
(Intercept)	0.20	0.21	0.13 – 0.30	**<0.001**
Improve Accuracy of Diagnostic Assessment	0.98	0.01	0.96 – 1.00	0.105
Experimental Condition [Networks]	0.53	0.37	0.25 – 1.09	0.084
Patient Demographic [Black female]	0.49	0.34	0.25 – 0.96	**0.037**
Improv. Accuracy Diag. Assessment * Experimental Condition [Networks]	1.05	0.02	1.01 – 1.09	**0.018**
Experimental Condition [Networks] * Patient Demographic [Black female]	3.45	0.51	1.27 – 9.38	**0.015**
Observations	577			
R^2 Tjur	0.026			

Supplementary Table 9: **Logistic regression examining the probability of clinicians improving their treatment recommendations as a function of (1) improvements in assessment accuracy, (2) patient demographic, and (3) experimental condition, while clustering standard errors at the trial level.** The data presented include only clinicians who initially provided the incorrect treatment recommendation (i.e., one of option A, B, or D), such that improvements in treatment recommendation is modeled as a binary variable where 1 indicates a clinician who revised from an incorrect to the guideline-recommended treatment recommendation by round 3, and 0 indicates a clinician who either did not revise their treatment recommendation, or otherwise revised their treatment recommendation to another incorrect option. All statistical comparisons are two-sided. Bolded p-values indicate statistical significance at the p<0.05 level.

Supplementary Table 9 presents a logistic regression examining the probability of clinicians improving in their treatment accuracy as a function of (1) improvements in clinicians' diagnostic

assessments, (2) patient demographic, and (3) experimental condition, while clustering standard errors at the trial level. First, we observe a significant effect of experimental condition on the relationship between improvements in the accuracy of diagnostic assessments and improvements in treatment quality. We find that the network condition is associated with a significantly higher probability that improvements in diagnostic accuracy correlate with improvements in treatment recommendations (p<0.05, OR=1.05, CI=[1.00, 1.09], n=577). This effect, importantly, holds while controlling for patient demographic. Furthermore, Supplementary Table 9 suggests that this effect is greater for the Black female patient than the white male patient (consistent with Figure 2*B*). Interacting the Black female patient with experimental conditions indicates a significantly higher probability of improving in treatment recommendation (p<0.01, OR=3.45, CI=[1.37, 9.38], n=577). Combined, these results suggest that the clinicians in social networks more consistently improved in the quality of their treatment recommendations, which is predicted by improvements in their diagnostic assessments. These results hold if either interaction term in Supplementary Table 9 is included in the model separately. Indeed, a reduced model shows that an interaction between improvements to diagnostic accuracy and experimental condition predicts that, in the network condition, improvements to diagnostic accuracy significantly correlate with improvements in treatment recommendation, while controlling for patient demographic (p=0.01, OR=1.05, CI=[1.01, 1.09], n=577).

	Probability of Guideline-Recommended Treatment (Final Recommendation)			
Predictors	*Odds Ratios*	*std. Error*	*CI*	*p*
(Intercept)	0.01	1.06	0.00 – 0.12	**<0.001**
Improve Diagnostic Accuracy	1.33	0.09	1.12 – 1.57	**0.001**
Patient Demographic [Black female]	2.60	0.88	0.47 – 14.53	0.277
Observations	83			
R^2 Tjur	0.272			

Supplementary Table 10: **Logistic regression examining the probability of providing the guideline-recommended final treatment recommendation for clinicians in the network condition who initially recommended unsafe undertreatment.** All standard errors are clustered at the trial level. All statistical comparisons are two-sided. Bolded p-values indicate statistical significance at the p<0.05 level.

Supplementary Table 10 displays the results of a logistic regression examining the probability of providing the guideline-recommended final treatment recommendation for clinicians in the network condition who initially recommended unsafe undertreatment. We find that improvements in clinicians' diagnostic assessments significantly predict an increase in the likelihood of clinicians changing their recommended treatment from unsafe undertreatment to the guideline-recommended treatment option (p<0.01, OR=1.33, CI=[1.12 – 1.57]).

	Guideline-Recommended Treatment							
	Control				Network			
Predictors	*Odds Ratios*	*std. Error*	*CI*	*p*	*Odds Ratios*	*std. Error*	*CI*	*p*
(Intercept)	0.15	0.19	0.10 – 0.22	**<0.001**	0.18	0.22	0.12 – 0.28	**<0.001**
Round of Recommendation	1.07	0.08	0.92 – 1.25	0.382	1.24	0.09	1.03 – 1.49	**0.021**
Patient Demographic [White male]	2.09	0.13	1.62 – 2.71	**<0.001**	0.81	0.15	0.61 – 1.08	0.155
Observations	1505				1151			
R^2	0.022				0.006			

Supplementary Table 11: **Logistic regression examining bias in treatment recommendations as a function of patient demographic, controlling for experimental condition and the round of recommendation in the experiment, while clustering standard errors at the trial level.** All statistical comparisons are two-sided. Bolded p-values indicate statistical significance at the p<0.05 level.

Supplementary Table 11 displays the logistic regression examining the effects of subsequent rounds of revision on the likelihood of clinicians providing the guideline-recommended clinical recommendation, while controlling for patient demographic and experimental condition, and while clustering standard errors at the trial level. In the control condition, we find that subsequent rounds of reflection had no effect on the likelihood of clinicians providing the guideline-recommended recommendation (p=0.38, OR=1.07, n=1,505); indeed, in the control condition, we find that baseline bias in favor of the white male patient persisted even when controlling for the number of rounds of reflection (p<0.001, OR=2.09, n=1,505). By contrast, in the network condition, we find that each round of revision with social influence is associated with a significant increase in the likelihood of providing the guideline-recommended clinical treatment (p=0.02, OR=1.24, n=1,151). Importantly, we find that this effect holds for both patients, when controlling for patient demographic (p=0.81, OR=0.15, n=1,151).

	Guideline Recommended Treatment			
Predictors	*Odds Ratios*	*std. Error*	*CI*	*p*
(Intercept)	0.28	0.15	0.21 – 0.38	**<0.001**
Round of Recommendation	1.14	0.06	1.01 – 1.28	**0.030**
Experimental Condition [Networks]	0.61	0.13	0.47 – 0.80	**<0.001**
Patient Demographic [Black female]	0.48	0.13	0.37 – 0.62	**<0.001**
Experimental Condition [Networks] * Patient Demographic [Black female]	2.59	0.20	1.76 – 3.82	**<0.001**
Observations	2656			
R^2 Tjur	0.015			

Supplementary Table 12: Logistic regression examining the interaction between experimental condition and patient demographic on the likelihood of providing the guideline-recommended clinical recommendation. Standard errors are clustered at the trial level. All statistical comparisons are two-sided. Bolded p-values indicate statistical significance at the p<0.05 level.

Supplementary Table 12 examines the interaction between experimental condition and patient demographic when predicting the likelihood of clinicians providing the guideline-recommended clinical recommendation. We find that social networks were particularly effective at increasing the likelihood of providing the guideline-recommended clinical recommendation for the Black female patient (p<0.001, OR=2.49, n=2,656). Comparing this model to the identical model without the interaction term indicates that the interaction term is a strong, significant predictor (χ^2 Test, p<0.001) of patient care. This individual-analysis corroborates the hypothesized effect of peer networks on reducing treatment-related biases in patient care, as shown in the trial-level results in Figure 1 in the main text.

	Unsafe Undertreatment			Undertreatment			Overtreatment		
Predictors	*Odds Ratios*	*CI*	*p*	*Odds Ratios*	*CI*	*p*	*Odds Ratios*	*CI*	*p*
(Intercept)	0.28 (0.18)	0.20 – 0.39	**<0.001**	1.24 (0.15)	0.94 – 1.66	**<0.001**	0.02 (0.38)	0.01 – 0.05	0.374
Patient Demographic [Black female]	1.25 (0.09)	1.04 – 1.51	0.178	1.07 (0.08)	0.92 – 1.25	0.597	0.93 (0.19)	0.64 – 1.35	0.697
Round of Recommendation	0.94 (0.08)	0.81 – 1.10	0.591	0.93 (0.06)	0.82 – 1.05	**0.021**	1.46 (0.16)	1.08 – 1.99	**0.045**
Experimental Condition [Networks]	1.80 (0.25)	1.10 – 2.93	**0.038**	0.69 (0.21)	0.45 – 1.05	**0.007**	3.12 (0.54)	1.09 – 8.96	0.086
Round of Recommendation * Experimental Condition [Networks]	0.76 (0.12)	0.60 – 0.96	**0.035**	1.23 (0.10)	1.01 – 1.49	**0.001**	0.51 (0.25)	0.31 – 0.83	**0.022**
Observations	2656			2656			2656		
R^2 Tjur	0.008			0.002			0.004		

Supplementary Table 13: **Logistic regression examining the effect of experimental condition on the likelihood of providing the remaining recommendation options (A, Unsafe Undertreatment; B, Undertreatment; D, Overtreatment).** Standard errors are clustered at the trial level and are shown in parentheses. All statistical comparisons are two-sided. Bolded p-values indicate statistical significance at the p<0.05 level.

Finally, in Supplementary Table 13 we examine the effect of the experimental treatment on the likelihood of clinicians recommending other clinical recommendations rather than the guideline-recommended treatment, namely: A, unsafe undertreatment; B, undertreatment; and D, overtreatment. Each model includes an interaction term between the round of the study (initial, second, and final recommendation), and the experimental condition (Control vs. Network). Across all incorrect clinical recommendations, we observe a significant effect of experimental condition. For option A (unsafe undertreatment), we find that in networks, subsequent rounds of revision led to a significant reduction in the likelihood of providing unsafe undertreatment (p=0.03, OR=0.76, n=2,656); the inclusion of the interaction term in the model is significant (χ^2 Test, p<0.05). Similarly, we find that in networks, subsequent rounds of revision led to a significant increase in the likelihood of providing option B, which indicates

an improvement in the quality-of-care relative to options A and D ($p<0.01$, OR=1.23, n=2,656); the inclusion of the interaction term in the model is significant (χ^2 Test, $p<0.05$). Lastly, we find that in networks, subsequent rounds of revision led to a significant decrease in the likelihood of providing option D (overtreatment) (p=0.02, OR=0.51, n=2,656); the inclusion of the interaction term in the model is significant (χ^2 Test, $p<0.05$).

	Magnitude of revision			
Predictors	*Estimates*	*std. Error*	*CI*	*p*
(Intercept)	1.04	0.79	-0.51 – 2.60	0.187
Intial Error Diagnostic Assessment	0.60	0.04	0.52 – 0.68	**<0.001**
Patient.Demographic [white male]	-1.34	0.93	-3.11 – 0.42	0.152
Observations	294			
R^2 / R^2 adjusted	0.446 / 0.442			

Supplementary Table 14: An OLS predicting the absolute magnitude of a clinician's revision to their diagnostic assessment (from initial to final assessment), as a function of clinicians' initial diagnostic error, while controlling for patient demographic and clustering standard errors at the trial. All data shown here are from the network condition. All statistical comparisons are two-sided. Bolded p-values indicate statistical significance at the p<0.05 level.

Supplementary Table 14 displays an OLS regression predicting the absolute magnitude of a clinician's revision to her/his diagnostic assessment (from initial to final assessment), as a function of their initial diagnostic error, while controlling for patient demographic and clustering standard errors at the trial level. All data shown here are from the network condition. As reported in the main text, we see that clinicians with greater initial error in their diagnostic assessments produce larger revisions to their diagnostic estimates as a result of social influence ($p<0.001$, r=0.66, SE=0.1, clustered by trial). This correlation holds equally for both the white male and Black female patient (p=0.15, r= -1.34, SE=0.93, clustered by trial).

Sensitivity Analyses

Robustness of Assessment Analysis. Based on prior research, our main analyses assume that a response of 16% is the most accurate diagnostic assessment for our vignette[7, 13]. Yet, the relevant medical literature concerning the cardiovascular symptoms described in our video vignette report a range of diagnostic assessments (12% - 17%) that may plausibly be considered accurate. Here we provide sensitivity analyses that demonstrate the robustness of our findings across this entire range of values.

First, we show that our main findings are robust for each of the individual values included in this range of possible assessment responses (12% - 17%).

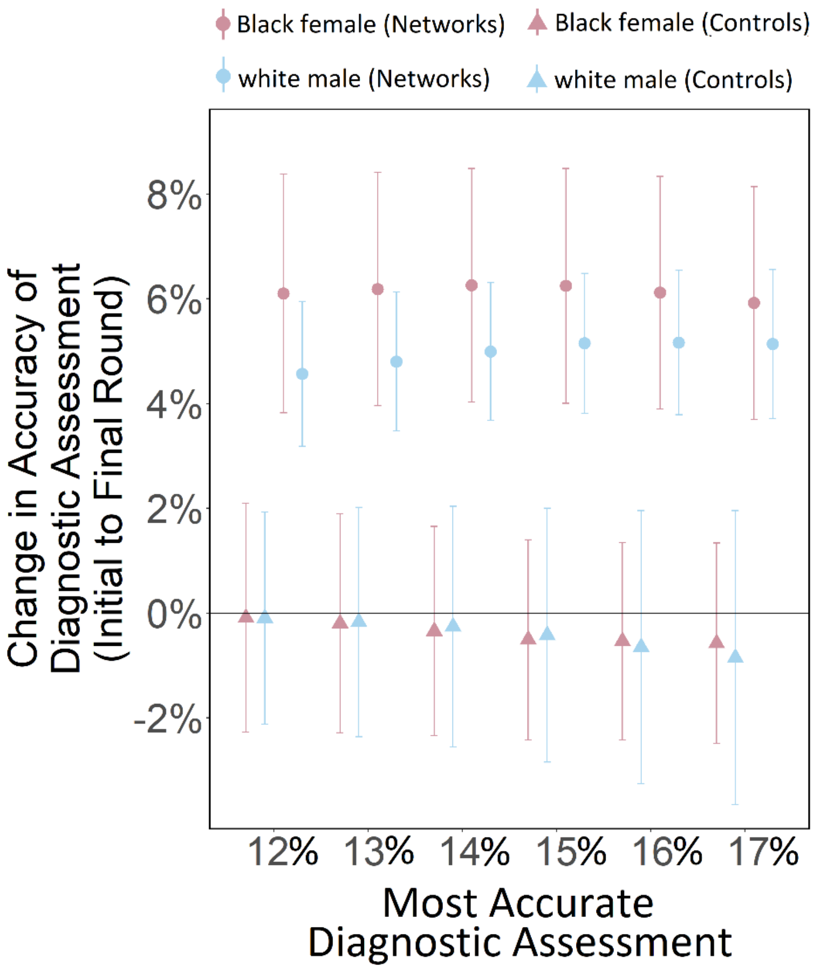

Supplementary Figure 9: The average change in accuracy of clinicians' diagnostic assessments (first to final round) for each patient in the control condition and network condition, as a function of the value identified as the most accurate diagnostic assessment. Results are first averaged within each trial in each condition, and then across trials (*N*=7) in each condition. 95% confidence intervals are shown.

Supplementary Figure 9 shows that there are no significant differences in our findings across the full range of assessment values that may be considered accurate. For each value in the acceptable range (12% - 17%), for both the Black female patient and the white male patient, clinicians in the network condition are significantly more likely to exhibit improved accuracy in their diagnostic assessments than clinicians in the control condition. Supplementary Figure 9 further illustrates that the overall change in diagnostic accuracy for clinicians in each condition is qualitatively similar

across the full range of assessment values. Importantly, this range of assessment values from 12%-17% constitute the peak values for assessment revision across all conditions. For values that fall outside of this range, e.g. below 12% and above 17%, the impact of the network revision process on clinicians' assessments decays rapidly to 0. Consistent with the analysis in the main text, we find that across all possible assessment values (0%-100%), the identified range, 12%-17%, corresponds to the primary set of values toward which clinicians in the network revise their assessments (exhibiting a maximum average change of 6%).

Correspondingly, and second, we assess an alternative approach to evaluating clinician accuracy that considers any response within the entire interval (12%-17%) to be an equally accurate response. Here we show that our main findings are robust to this "window" approach to evaluating assessment accuracy. In this analysis, for any assessment x, the error of x is measured as 0 if $12 \leq x \geq 17$. Accordingly, the error of any assessment $x < 12$ is measured as abs($12 - x$), and the error of any assessment $x > 17$ is measured as abs($17 - x$). Thus, an assessment value of 11% is considered to be 1 percentage point below the accurate response, and an assessment value of 18% is considered to be 1 percentage point above the accurate response. Consistent with the analytical approach used in the main text, we use min-max normalization to present the results in terms of overall changes (from the initial assessment to the final assessment) in the accuracy of clinicians' assessments, reporting both patients in both conditions. Supplementary Figure 10 shows that this window approach to evaluating assessment accuracy does not significantly alter our main results.

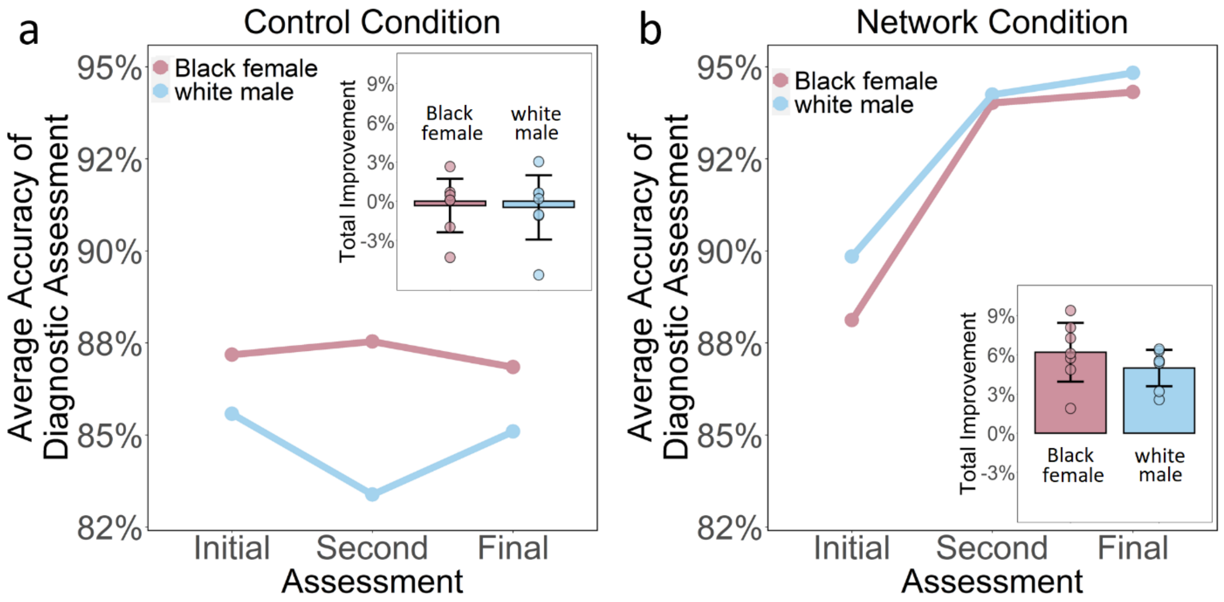

Supplementary Figure 10: Changes in clinicians' diagnostic assessments in the control and network condition, assuming a windowed approach to defining the most accurate diagnostic assessment. Panels A and B show the change (from the initial assessment to the final assessment) in clinicians' average diagnostic accuracy. Diagnostic accuracy is measured using a "window" approach for the complete range 12%-17%, such that for any assessment x in which x is less than 12 the error of this assessment is measured as abs($12 - x$); similarly, the error of any assessment x in which x is more than 17 is measured as abs($17 - x$). Results are first averaged within each trial in each condition, and then across trials (N=7) in each condition. The insets in both panels show the total improvement (in percentage points) for the accuracy of clinicians' assessments. Error bars display 95% confidence intervals; data points in panel insets display the mean improvement for each trial.

Consistent with the results reported in the main text, in the control condition we observe no significant changes in clinicians' diagnostic assessments as a result of independent revisions. By contrast, in the network condition clinicians exhibited significant improvements in the accuracy of their diagnostic assessments, both for the Black female patient ($p<0.01$) and the white male patient ($p<0.01$) (Wilcoxon Signed Rank Test).

Robustness of Recommendation Analysis. For the patient vignette used in this study, option C is the guideline-recommended option, and therefore the highest standard of care. However, option B (undertreatment) may also be considered acceptable care since it is a significantly better clinical choice than either option A (unsafe undertreatment) or option D (overtreatment). Here we evaluate the sensitivity of our findings to an analytical approach that treats both option B and option C as acceptable clinical recommendations. Our results show that our main findings are robust to the inclusion of both option B and option C.

First, we show that if we consider both options B and C to be acceptable, our main findings for the relative changes in quality of care across experimental conditions are qualitatively similar to the findings in the main text.

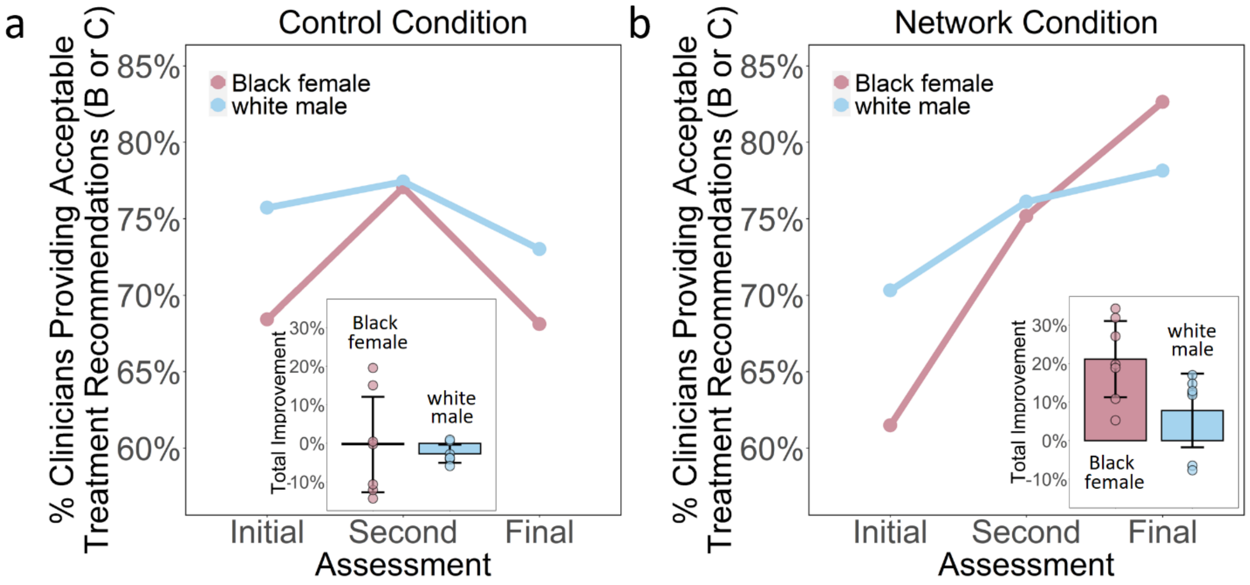

Supplementary Figure 11: Changes in the quality of clinicians' treatment recommendations in the control and network condition, while treating both option B and C as acceptable options. Panels A and B show the change (from the initial recommendation to the final recommendation) in the proportion of clinicians providing the white male and Black female patients with an acceptable treatment recommendation – either Option B, daily 81mg aspirin and stress test within two to three days (i.e., undertreatment), or Option C, referral to the emergency department for cardiac evaluation (i.e., guideline-recommended care). Panel A shows control conditions. Panel

B shows network conditions. Results are first averaged within each trial in each condition, and then across trials (*N*=7) in each condition. The insets in both panels show the total improvement (in percentage points) in the percent of clinicians recommending acceptable treatment (B or C). Error bars display 95% confidence intervals; data points in panel insets display the mean improvement for each trial.

Consistent with the analysis in the main text, initially all clinicians were significantly more likely to provide an acceptable clinical recommendation (option B or option C) to the white male patient (73% of responses) than the Black female patient (65% of responses) (p=0.01, χ^2=6.02). After two rounds of revision in the control condition, there was no significant change in the quality of clinicians' treatment recommendations, for either the white male patient (p>0.08, n=7, Supplementary Figure 11*A* inset, Wilcoxon Signed Rank Test, Two-sided) or the Black female patient (p>0.9, n=7, Supplementary Figure 11*A* inset, Wilcoxon Signed Rank Test, Two-sided). By contrast, Supplementary Figure 11*B* shows that in the network condition there was a significant improvement (from the initial treatment recommendation to the final treatment recommendation) in the recommendations given to the Black female patient (p=0.01, n=7 observations, Wilcoxon Signed Rank Test, Two-sided; Supplementary Figure 11*B* inset); consistent with the findings in the main text, there was no significant change in the treatment recommendations given to the white male patient (p=0.08, n=7 observations, Wilcoxon Signed Rank Test, Two-sided; Supplementary Figure 11*B* inset).

Second, we evaluate the robustness of our analysis of the effects of experimental conditions on the odds of clinicians recommending unsafe undertreatment (option A) versus an acceptable treatment (assuming both option B and option C are considered acceptable treatments) to both patients. We find similar results to those reported in the main text. As shown below in Supplementary Figure 12, we find that for an initially large inequity of care between the white male patient and Black female patient, the network condition produces a significant reduction in

the relative odds of clinicians' recommending unsafe undertreatment (option A) rather than an acceptable treatment (option B or option C) for the Black female patient.

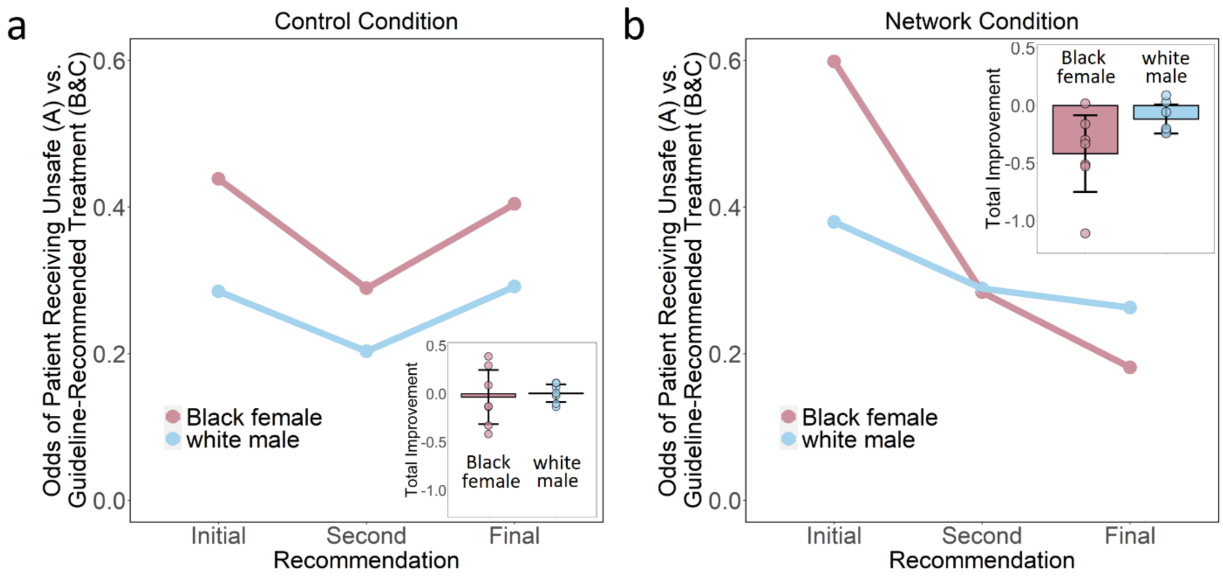

Supplementary Figure 12: **Changes in the inequity of clinicians' treatment recommendations in the control and network condition, while treating both option B and C as acceptable options.** Panels A and B show the change (from the initial response to the final response) in the odds of clinicians recommending option A (unsafe undertreatment) rather than recommending either option B or option C, for both patients. Panel A shows the control conditions. Panel B shows the network conditions. Results are first averaged within each trial in each condition, and then across trials (*N*=7) in each condition. The insets in both panels show the total change in the odds ratio of clinicians recommending unsafe undertreatment rather than either of the acceptable treatments, for each patient. Error bars display 95% confidence intervals; data points in panel insets display the mean improvement for each trial.

Consistent with the analyses in the main text, in the control condition after two rounds of revision, there were no significant changes in the odds of clinicians recommending unsafe undertreatment (option A) rather than an acceptable treatment (option B or option C), for either the white male patient (*p*=0.68, *n*=7, Wilcoxon Signed Rank Test, Two-sided) or the Black female patient (*p*=0.93, *n*=7, Wilcoxon Signed Rank Test, Two-sided). By contrast, in the network condition there was a significant reduction in the odds of clinicians recommending unsafe

undertreatment (option A) rather than an acceptable treatment (option B or option C) (*p*<0.05, *n*=7, Wilcoxon Signed Rank Test, Two-sided) for the Black female patient. Consistent with the analysis in the main text, there was no significant reduction in clinicians' odds of recommending unsafe undertreatment (option A) rather than an acceptable treatment (option B or option C) (*p*=0.10, *n*=7, Wilcoxon Signed Rank Test, Two-sided) for the white male patient.

Robustness to Attrition. Our main results are reported using an intention-to-treat sample, which includes all clinicians for each condition, regardless of how many rounds for which they responded (Supplementary Figure 1). Here we show that our results are robust to excluding clinicians who exhibited attrition by failing to complete all rounds of the task.

When comparing our sample with attrition excluded to our intention-to-treat sample, we do not observe any statistically significant differences in the percent of clinicians who provided the guideline-recommended treatment for the white male patient in the control condition (*p*=0.84, *n*=14, Wilcoxon Rank Sum Test, Two-sided), the Black female patient in the control condition (*p*=0.85, *n*=14, Wilcoxon Rank Sum Test, Two-sided), the white male patient in the network condition (*p*=0.53, *n*=14, Wilcoxon Rank Sum Test, Two-sided), and the Black female patient in the network condition (*p*=0.65, *n*=14, Wilcoxon Rank Sum Test, Two-sided). Similarly, when comparing our sample with attrition excluded to our intention-to-treat sample, we do not observe any statistically significant differences in the change in percent of clinicians providing the guideline-recommended treatment for the white male patient in the control condition (*p*=0.12, *n*=14, Wilcoxon Rank Sum Test, Two-sided), the Black female patient in the control condition (*p*=0.71, *n*=14, Wilcoxon Rank Sum Test, Two-sided), the white male patient in the network condition (*p*=0.7, *n*=14, Wilcoxon Rank Sum Test, Two-

sided), and the Black female patient in the network condition (p=0.62, n=14, Wilcoxon Rank Sum Test, Two-sided).

Robustness to Clinician Traits. Here we provide evidence that the demographic traits among clinicians in our sample did not interact with our experimental treatment as an explanation of the effects reported (Supplementary Table 1). When using logistic regression to predict baseline accuracy among clinicians, we find that clinicians were significantly more likely to recommend the guideline-recommended treatment to the white male patient than the Black female patient, even when controlling for the gender and age of clinicians, along with whether clinicians operate in a private practice and in primary care capacity (p<0.001, OR=4.7, n=288). We observed that primary care clinicians were mildly associated with lower likelihood of providing the guideline-recommended treatment at baseline (p=0.04, OR=0.45). All other demographic traits held no significant relationship with the quality of baseline treatment recommendations, and no traits were sufficient to account for the effect of patient demographic on the baseline accuracy of clinicians. In addition, we found that none of the demographic traits above correlated significantly with the likelihood that clinicians' treatment recommendations improved, either in the control or the network condition.

Supplementary References

1. Becker, J., Brackbill, D. & Centola, D. Network dynamics of social influence in the wisdom of crowds. *PNAS* **114**, E5070–E5076 (2017).
2. Becker, J., Porter, E. & Centola, D. The wisdom of partisan crowds. *PNAS* **116**, 10717–10722 (2019).
3. Guilbeault, D., Becker, J. & Centola, D. Social learning and partisan bias in the interpretation of climate trends. *PNAS* **115**, 9714–9719 (2018).

4. Guilbeault, D. & Centola, D. Networked collective intelligence improves dissemination of scientific information regarding smoking risks. *PLoS One* **15**, e0227813 (2020).
5. Maslov, S., & Sneppen, K. Specificity and stability in topology of protein networks. *Science* **296**, 910–13 (2002).
6. Mahler SA, et al. The HEART Pathway randomized trial: identifying emergency department patients with acute chest pain for early discharge. *Circ Cardiovasc Qual Outcomes* **8**, 195-203 (2015).
7. Backus BE*, et al.* A prospective validation of the HEART score for chest pain patients at the emergency department. *Int J Cardiol* **168**, 2153-2158 (2013).
8. Byrne, C., Toarta, C., Backus, B., Holt, T. The HEART score in predicting major adverse cardiac events in patients presenting to the emergency department with possible acute coronary syndrome: protocol for a systematic review and meta-analysis. *Syst Rev*. **7**, 148 (2018).
9. Ebell MH. Evaluation of chest pain in primary care patients. *Am Fam Physician* **83**, 603-605 (2011).
10. Poldervaart JM, Reitsma JB, Backus BE, Koffijberg H, Veldkamp RF, Ten Haaf ME. Effect of using the HEART score in patients with chest pain in the emergency department. *Ann Intern Med* **167**, 141-141 (2017).
11. Noether, G. E. Sample size determination for some common nonparametric tests. *J Am Stat Assoc* **82**, 645–647 (1987).
12. Andridge, R. R. & Little, R. J. A. A review of Hot Deck Imputation for survey non-response. *Int Stat Rev* **78**, 40–64 (2010).
13. Gomez, R. HEART score for predicting adverse outcomes in patients with chest pain. *Am Fam Physician* **98**, 72-75 (2018).
