# Supplementary material for: The reduction of race and gender bias in clinical treatment recommendations using clinician peer networks in an experimental setting
Source: Nat Commun. 2021 Nov 15;12:6585. doi: 10.1038/s41467-021-26905-5 (PMC8593068; doi:10.1038/s41467-021-26905-5)
Supplement: Supplementary file 2 — Reporting summary [file 41467_2021_26905_MOESM2_ESM.pdf]

nature research

Corresponding author(s): Damon Centola

Last updated by author(s): Oct 20, 2021

Reporting Summary

Nature Research wishes to improve the reproducibility of the work that we publish. This form provides structure for consistency and transparency in reporting. For further information on Nature Research policies, see our Editorial Policies and the Editorial Policy Checklist.

Statistics

For all statistical analyses, confirm that the following items are present in the figure legend, table legend, main text, or Methods section.

n/a	Confirmed	
☐	☒	The exact sample size (*n*) for each experimental group/condition, given as a discrete number and unit of measurement
☐	☒	A statement on whether measurements were taken from distinct samples or whether the same sample was measured repeatedly
☐	☒	The statistical test(s) used AND whether they are one- or two-sided<br>*Only common tests should be described solely by name; describe more complex techniques in the Methods section.*
☐	☒	A description of all covariates tested
☐	☒	A description of any assumptions or corrections, such as tests of normality and adjustment for multiple comparisons
☐	☒	A full description of the statistical parameters including central tendency (e.g. means) or other basic estimates (e.g. regression coefficient) AND variation (e.g. standard deviation) or associated estimates of uncertainty (e.g. confidence intervals)
☐	☒	For null hypothesis testing, the test statistic (e.g. *F*, *t*, *r*) with confidence intervals, effect sizes, degrees of freedom and *P* value noted<br>*Give P values as exact values whenever suitable.*
☐	☒	For Bayesian analysis, information on the choice of priors and Markov chain Monte Carlo settings
☐	☒	For hierarchical and complex designs, identification of the appropriate level for tests and full reporting of outcomes
☒	☐	Estimates of effect sizes (e.g. Cohen's *d*, Pearson's *r*), indicating how they were calculated

Our web collection on statistics for biologists contains articles on many of the points above.

Software and code

Policy information about availability of computer code

Data collection	The data were collected using a proprietary mobile app called "The DxChallenge."
Data analysis	The code used in analyzing the data in this study were custom built in R. All code relevant for the reproduction of our statistical analyses can be found here: https://github.com/drguilbe/cliniciansCI or https://ndg.asc.upenn.edu/ See the "Code Availability" statement in the manuscript for further details.

For manuscripts utilizing custom algorithms or software that are central to the research but not yet described in published literature, software must be made available to editors and reviewers. We strongly encourage code deposition in a community repository (e.g. GitHub). See the Nature Research guidelines for submitting code & software for further information.

Data

Policy information about availability of data

All manuscripts must include a data availability statement. This statement should provide the following information, where applicable:

- Accession codes, unique identifiers, or web links for publicly available datasets
- A list of figures that have associated raw data
- A description of any restrictions on data availability

Data Availability: All data relevant for the reproduction of our statistical analyses can be found here: https://github.com/drguilbe/cliniciansCI or https://ndg.asc.upenn.edu/ See the Data Availability" statement in the manuscript for further details.

Field-specific reporting

Please select the one below that is the best fit for your research. If you are not sure, read the appropriate sections before making your selection.

☐ Life sciences ☒ Behavioural & social sciences ☐ Ecological, evolutionary & environmental sciences

For a reference copy of the document with all sections, see nature.com/documents/nr-reporting-summary-flat.pdf

Behavioural & social sciences study design

All studies must disclose on these points even when the disclosure is negative.

Study description	This is a quantitative, experimental study of how peer consultation networks among clinicians impact the accuracy of their diagnostic assessments and treatment recommendations, as a function of patient demographic.
Research sample	Baseline characteristics did not differ significantly between the two groups except for the date of NPI assignment, with more clinicians with NPI assignments in 2009-2012 assigned to the control condition (Table 1). Fig. S2 displays the geographical location of the clinicians that made up the recruitment pool for this study. These details are copied here. Network condition: Male (62.4%), Primary Care (91.6%), Independent Practice (23.4%), Date of NPI License (2005-2008: 10.3%, 2009-2012: 7%, 2013-2016: 32.8%, 2017-present: 49.8%); Control condition: Male (64.8%), Primary Care (87.0%), Independent Practice (21.9%), Date of NPI License (2005-2008: 8.6%, 2009-2012: 13.6%, 2013-2016: 32.2%, 2017-present: 45.4%). This sample is not nationally representative. This sample was gathered as a convenience sample. Please see "sampling strategy" below.
Sampling strategy	Clinicians were recruited from around the US by distributing advertisements over clinician discussion boards on Reddit and Facebook's advertising platforms. Seven recruitment advertisements were posted on Reddit, specifically on messaging boards that attract doctors and resident clinicians. We distributed three advertisements over Facebook, from March to November 2019, while making use of Facebook's advertising platform to target clinicians. We limited advertisement exposure to people who resided in the US, who were 18 to 65, and whose demographic characteristics were among the following features suggested by Facebook: doctor (Dr), medical doctor (MD), and medical director (MD). Beyond online recruitment, clinicians were also recruited through Penn Medicine's Graduate Medical Education training program (for resident MD clinicians). Advertisements were circulated to the 2017 cohort of resident clinicians, and clinicians were also recruited through outreach events as part of Penn Medicine's orientation for incoming residents. Our sample procedure attempted to maximize the available sample size for our experiment, given uncertainty regarding the anticipated effect size. However, effect sizes from prior studies suggested that, assuming a strong effect size, a sample of 7 trials in each experimental condition would provide the minimal lower bound required to anticipate a treatment effect with 80% power.
Data collection	To initiate a trial, the app sent push notifications to all 1100 clinicians who had registered for the study (Fig. S3). Once 120 clinicians had responded, they were randomized to conditions in a 2:1 ratio – 80 clinicians were randomized to the intervention conditions, and 40 clinicians were randomized to the control conditions (Fig. S1). The 80 clinicians randomized to the network condition were then randomized in a 1:1 ratio into each of the network conditions (white male patient or black female patient). The 40 clinicians in the control condition were then randomized in a 1:1 ratio into each of the control conditions (white male patient or black female patient). The researchers collected the data were not blind to the research hypotheses. However, all randomizations were automated through the DxChallenge app, such that the experimenters were blind to the random assignments of clinicians to condition. DG and JZ were present for the data collection.
Timing	From March 1, 2017 to November 29, 2019, we recruited 1100 clinicians of whom 840 responded (560 network, 260 control) (Fig. S1) to one of the push notifications for this study (Fig. S1).
Data exclusions	No data were excluded from this study. Our analyses were calculated using the intention-to-treat sample (see Fig. S1).
Non-participation	14% of participants who entered the game exhibited attrition across trials. Several factors may account for this attrition. One possible factor is that the clinician participants in our sample may have been unexpectedly unable to participate or complete the DxChallenge task as a result of responsibilities and demands in their clinical workplace.
Randomization	To initiate a trial, the app sent push notifications to all 1100 clinicians who had registered for the study (Fig. S3). Once 120 clinicians had responded, they were randomized to conditions in a 2:1 ratio – 80 clinicians were randomized to the intervention conditions, and 40 clinicians were randomized to the control conditions (Fig. S1). The 80 clinicians randomized to the network condition were then randomized in a 1:1 ratio into each of the network conditions (white male patient or black female patient). The 40 clinicians in the control condition were then randomized in a 1:1 ratio into each of the control conditions (white male patient or black female patient). All randomizations were automated through the app. (See "Statistical Analyses" for greater detail).

Reporting for specific materials, systems and methods

We require information from authors about some types of materials, experimental systems and methods used in many studies. Here, indicate whether each material, system or method listed is relevant to your study. If you are not sure if a list item applies to your research, read the appropriate section before selecting a response.

Materials & experimental systems

n/a	Involved in the study	
☒	☐	Antibodies
☒	☐	Eukaryotic cell lines
☒	☐	Palaeontology and archaeology
☒	☐	Animals and other organisms
☐	☒	Human research participants
☒	☐	Clinical data
☒	☐	Dual use research of concern

Methods

n/a	Involved in the study	
☒	☐	ChIP-seq
☒	☐	Flow cytometry
☒	☐	MRI-based neuroimaging

Human research participants

Policy information about studies involving human research participants

Population characteristics	Baseline characteristics did not differ significantly between the two groups except for the date of NPI assignment, with more clinicians with NPI assignments in 2009-2012 assigned to the control condition (Table 1). Fig. S2 displays the geographical location of the clinicians that made up the recruitment pool for this study. These details are copied here. Network condition: Male (62.4%), Primary Care (91.6%), Independent Practice (23.4%), Date of NPI License (2005-2008: 10.3%, 2009-2012: 7%, 2013-2016: 32.8%, 2017-present: 49.8%); Control condition: Male (64.8%), Primary Care (87.0%), Independent Practice (21.9%), Date of NPI License (2005-2008: 8.6%, 2009-2012: 13.6%, 2013-2016: 32.2%, 2017-present: 45.4%). No direct information on clinicians' age or ethnicity was available.
Recruitment	Clinicians were recruited from around the US by distributing advertisements over clinician discussion boards on Reddit and Facebook's advertising platforms. Seven recruitment advertisements were posted on Reddit, specifically on messaging boards that attract doctors and resident clinicians. We distributed three advertisements over Facebook, from March to November 2019, while making use of Facebook's advertising platform to target clinicians. We limited advertisement exposure to people who resided in the US, who were 18 to 65, and whose demographic characteristics were among the following features suggested by Facebook: doctor (Dr), medical doctor (MD), and medical director (MD). Beyond online recruitment, clinicians were also recruited through Penn Medicine's Graduate Medical Education training program (for resident MD clinicians). Advertisements were circulated to the 2017 cohort of resident clinicians, and clinicians were also recruited through outreach events as part of Penn Medicine's orientation for incoming residents. While our recruitment strategy cannot rule out selection effects from those clinicians who were willing to participate in our app-based experiment, we control for potential selection effects by recruiting from separate platforms, and by blinding all clinician participants to the (i) design and purpose of the experiment, and (ii) to the randomization process determining their participation in a particular experimental condition. We maintain that an important direction for future research is to evaluate whether our results generalize across a representative sample of clinicians in the U.S. and in other national contexts.
Ethics oversight	This research was approved by the Institutional Review Board at the University of Pennsylvania, where the study was conducted, and it included informed consent by all participants in the study.

Note that full information on the approval of the study protocol must also be provided in the manuscript.
